# A prognostic lncRNA signature associated with ribonucleotide reductase predicts overall survival and immune landscape in hepatocellular carcinoma

**DOI:** 10.3389/fcell.2026.1908145

**Published:** 2026-08-13

**Authors:** Kepu Zheng, Haitao Jiang, Yanghui Wen, Jianjian Xiang, Wuke Wang, Jie Wang, Zhilin Liu, Yu Tang, Yunjie Chen, Leiyang Dai, Feng Ren, Xiang Huang

**Affiliations:** 1 Department of Hepato-Biliary-Pancreatic Surgery, Ningbo No. 2 Hospital, Wenzhou Medical University, Ningbo, Zhejiang, China; 2 Department of Genetics, Zunyi Medical University, Zunyi, Guizhou, China; 3 Department of Clinical Laboratory, The First Affiliated Hospital of Kunming Medical University, Kunming, China; 4 Department of Medical Experiment, Ningbo No. 2 Hospital, Wenzhou Medical University, Ningbo, Zhejiang, China

**Keywords:** hepatocellular carcinoma (HCC), immunotherapy response, prognosis, ribonucleotide reductase, survival analysis

## Abstract

**Introduction:**

Hepatocellular carcinoma (HCC) is a major cause of cancer‐related mortality, and effective prognostic stratification remains urgently needed. Ribonucleotide reductase (RR), consisting mainly of RRM1 and RRM2, plays a critical role in nucleotide metabolism and tumor progression. This study aimed to develop an RR‐related long non‐coding RNA (lncRNA) signature for predicting prognosis and exploring potential therapeutic implications in HCC.

**Methods:**

Transcriptomic and clinical data from 377 HCC patients in The Cancer Genome Atlas (TCGA) database were analyzed. Molecular subgroups were identified based on RRM1 and RRM2 expression. Differentially expressed RR‐related lncRNAs were screened, and an RR‐indexed lncRNA prognostic signature (RILPS) was established using univariate Cox and LASSO Cox regression analyses. A nomogram integrating RILPS and M stage was constructed, followed by analyses of immune infiltration, tumor mutation burden (TMB), microsatellite instability (MSI), pathway enrichment, and drug sensitivity.

**Results:**

Two distinct RRM1/RRM2‐associated molecular clusters were identified. A 65‐lncRNA‐based RILPS model was developed and effectively stratified HCC patients into different prognostic groups. The RILPS‐M stage nomogram showed favorable predictive performance, with AUC values of 0.767, 0.800, and 0.820 for 1‐, 3‐, and 5‐year overall survival, respectively. The high‐RILPS group exhibited increased immune infiltration, higher TMB, and MSI characteristics, whereas the low‐RILPS group was enriched in metabolic pathways. Key lncRNAs, including NBAT1, LINC01138, and LINC00671, were associated with HCC prognosis and may contribute to tumor progression. Drug sensitivity analysis suggested potential therapeutic differences between RILPS‐defined subgroups.

**Discussion:**

The RR‐related lncRNA signature integrates prognostic, immune, and metabolic features in HCC. RILPS may serve as a promising tool for risk stratification and personalized treatment guidance, while the identified lncRNAs provide potential candidates for further mechanistic investigation.

## 1 Introduction

Hepatocellular carcinoma (HCC) is a primary liver cancer with a high mortality rate and represents the predominant histological subtype among liver cancers (Craig et al., 2020). The occurrence and development of HCC are influenced by many factors, such as chronic hepatitis B, chronic hepatitis C, alcohol consumption, smoking, diabetes, and obesity (Vogel et al., 2022; Koulouris et al., 2021). These factors may contribute to the onset and progression of HCC by influencing gene expression and cell signal transduction pathways in liver cells. The treatment of HCC includes surgical resection, liver transplantation, radiotherapy, chemotherapy and targeted therapy (Vogel et al., 2022; Maluccio and Covey, 2012). Liver cancer is often diagnosed at an advanced stage and has a poor prognosis due to its high rate of recurrence and metastasis. Therefore, early screening and diagnosis become the key to improve the survival rate of HCC. It is crucial to construct a prognostic signature for HCC to delve deeper into its pathogenesis and explore more effective treatment options.

Cell proliferation is a fundamental process in organism growth, development and maintenance of tissue homeostasis (Zhu and Thompson, 2019). The progression of HCC is closely associated with abnormal cell proliferation (Garcia-Lezana et al., 2021). A close relationship has been identified between cell proliferation and ribonucleotide reductase (RR), an essential enzyme in nucleotide metabolism that acts as a rate-limiting factor. RR facilitates the conversion of ribonucleotides to deoxyribonucleotides, playing a vital role in DNA synthesis and repair (Aye et al., 2014). It thus play crucial roles in regulating cell proliferation and differentiation. RR exists mainly in the form of heterodimeric tetramer, with large catalytic subunit M1 (RRM1) and small regulatory subunit M2 (RRM2) (Aye et al., 2014). Although the RRM2 subunit is regulated in a cell-cycle specific manner, the expression of RRM1 remains relatively constant in actively proliferating cells (Freeman and genetics, 2011). Importantly, the enzymatic activity of RR depends on the coordinated interaction between RRM1 and RRM2, as neither subunit alone is sufficient to catalyze the reduction of ribonucleotides into deoxyribonucleotides. The dynamic balance between the catalytic activity of RRM1 and the cell cycle-dependent regulation of RRM2 is essential for maintaining intracellular deoxyribonucleotide pools, genome stability, and DNA replication fidelity. Dysregulation of this coordinated system may promote uncontrolled cell proliferation and tumor progression. However, despite increasing evidence regarding the individual roles of RRM1 and RRM2 in HCC, their coordinated regulatory mechanisms and downstream molecular networks remain poorly understood.

So far, research has demonstrated the association of RRMs with various types of cancer, including liver cancer (Yang et al., 2020), lung cancer (Zhu et al., 2018; Ma et al., 2020; Chen et al., 2018), and breast cancer (Abdel-Rahman et al., 2022). Specifically, in HCC, accumulating evidence has revealed the expression patterns and functional roles of RRMs. Most studies have reported that RRM2 is significantly upregulated in HCC tissues compared with adjacent non-tumor tissues, and elevated RRM2 expression is closely associated with unfavorable prognosis in HCC patients (Lee et al., 2014; Qin et al., 2023; Ibrahim et al., 2025). Mechanistically, RRM2 has been shown to promote HCC cell proliferation, migration, and invasion by facilitating DNA synthesis and regulating cell cycle progression (Wu et al., 2024; Li et al., 2024). In addition, emerging evidence suggests that RRM2 may be involved in resistance to therapeutic agents such as 5-fluorouracil, possibly through maintaining nucleotide supply and facilitating DNA damage repair (Zhan et al., 2021; Tong et al., 2024; Zhou et al., 2022). Similarly, RRM1 has also been found to be upregulated in tumor tissues and associated with aggressive tumor behavior and poor prognosis in HCC (Mao et al., 2022; Yin et al., 2023). Indeed, accumulating evidence in HCC supports RRM1 as a pro-tumorigenic factor, where elevated RRM1 expression is associated with enhanced tumor proliferation, invasion, therapeutic resistance, and unfavorable clinical outcomes. However, its biological functions appear to vary across cancer types, as RRM1 has been implicated in tumor progression and therapeutic resistance in some malignancies while exhibiting tumor-suppressive properties in others (Jordheim et al., 2011; Zheng et al., 2007; Lin et al., 2024). These apparently divergent observations suggest that the biological functions of RRM1 are highly context-dependent and may be influenced by tissue-specific molecular regulatory networks. Therefore, a systematic investigation of the regulatory mechanisms governing RRM1, particularly its associated lncRNA network in HCC, is warranted to better clarify its context-specific roles in hepatocarcinogenesis. Despite these findings, the exact functional differences and coordinated regulatory mechanisms of RRM1 and RRM2 in HCC progression remain incompletely understood.

Long non-coding RNAs (lncRNAs) are a class of non-protein-coding RNA transcripts longer than 200 nucleotides. Although they do not encode proteins, lncRNAs play critical regulatory roles in gene expression at the epigenetic, transcriptional, and post-transcriptional levels. They are involved in various biological processes and contribute to tumor initiation and progression (Noh et al., 2018; Qiu et al., 2021). Studies have pointed out that LncRNAs can be used as potential diagnostic markers and new drug targets (Salimian et al., 2023; Heydari et al., 2024). Importantly, the regulatory relationship between RRMs and lncRNAs has been increasingly explored in various cancers. For example, in non-small cell lung cancer, lncRNAs can regulate RRM2 expression via ceRNA mechanisms, thereby promoting tumor cell proliferation (Yang et al., 2019). The lncRNA lincNMR has been reported to promote cancer progression by regulating nucleotide metabolism through a YBX1–RRM2 axis (Gandhi et al., 2020), highlighting the involvement of RRM2-related pathways in lncRNA-mediated tumor regulation. In HCC, RRM1 expression has been shown to be regulated by lncRNA-mediated ceRNA networks, including the MIR4435-2HG/miR-22-3p and SNHG6/miR-101-3p axes (Mao et al., 2022). In recent years, an increasing number of studies have developed lncRNA-based prognostic signatures for HCC, particularly those associated with tumor immunity, metabolic reprogramming, ferroptosis, and other cancer-related biological processes (Zhan et al., 2023; Yu et al., 2025; Zheng et al., 2024; Mei et al., 2024). These studies have highlighted the important prognostic and therapeutic relevance of lncRNA regulatory networks in HCC progression. Despite these studies, the lncRNA–RRM regulatory network in HCC has not been systematically characterized, and the prognostic value of RRM-associated lncRNAs, particularly in relation to tumor immunity and metabolic reprogramming, remains largely unclear. Therefore, in this study, we aimed to systematically identify RRM-associated lncRNAs and construct a prognostic signature based on TCGA data. Furthermore, we explored the relationships between the risk model and tumor immunity, metabolic pathways, and drug sensitivity, in order to provide new insights into the molecular mechanisms and potential therapeutic strategies for HCC.

## 2 Materials and methods

### 2.1 HCC dataset acquisition and preprocessing

HCC RNA-seq count data, including tumor samples and paired adjacent non-tumor tissues, together with corresponding clinical information, were retrieved from the TCGA online database (https://portal.gdc.cancer.gov/) via the “TCGAbiolinks” R package (v. 2.27.4) (Colaprico et al., 2016) in R software (v. 4.3.0). Data retrieval was performed on 21 February 2024, corresponding to GDC Data Release 38.0. The search command used was: GDCquery (project = “TCGA-LIHC”, legacy = FALSE, experimental. strategy = “RNA-Seq”, data. category = “Transcriptome Profiling”, data. type = “Gene Expression Quantification”, workflow. type = “STAR-Counts”). The distribution of patients by pathologic stage was as follows: 175 in Stage I, 87 in Stage II, 86 in Stage III, 5 in Stage IV, and 24 with unknown stage information.

The TCGA data were randomly divided into training and test cohorts at a 3:2 ratio using R software. The expression levels of 4,285 LncRNAs were extracted for analysis. Immunohistochemical (IHC) images of normal and HCC tissues were obtained from the Human Protein Atlas (HPA) (https://www.proteinatlas.org/).

### 2.2 Identification of differentially expressed LncRNAs

We obtained human LncRNA annotation information using GENCODE (version 43) (Frankish et al., 2023), and LncRNA expression data were extracted from HCC RNA-Seq datasets. Consensus clustering was conducted using the “ConsensusClusterPlus” R package to categorize the TCGA samples into two groups, namely cluster 1 and cluster 2, based on the expression patterns of RRM1 and RRM2 (MD and Bioinformatics, 2010). Subsequently, differentially expressed lncRNAs (DE-lncRNAs) between cluster 1 and cluster 2 were analyzed using the “DESeq2” package based on count data (v. 1.42.0; p < 0.05, |log2foldchange| > 0.5) (Love et al., 2014).

### 2.3 Establishment and validation of RILPS

First, univariate Cox regression analysis was conducted on obtained DE-lncRNAs to screen prognostic LncRNAs (p < 0.05). Subsequently, the prognostic LncRNAs were subjected to LASSO-penalized Cox regression analysis. Ten-fold cross-validation was performed to determine the optimal tuning parameter λ. The λ_min value, corresponding to the minimum cross-validated error, was selected as the optimal penalty parameter. This procedure yielded 65 lncRNAs with non-zero coefficients, which were used to construct the RRM-indexed LncRNA prognostic signature (RILPS). The coefficient of each factor was multiplied by the corresponding gene expression. Eventually, all the values were summed up. The risk score was calculated using the following formula:

$$RILPS=\sum_{i=1}^{n}Coef\left(i\right)\times \mathrm{Exp}\left(i\right)$$

### 2.4 Nomogram construction

To enhance clinical utility, a nomogram was established based on M-stage and RILPS (Iasonos et al., 2008). A calibration curve was constructed to show the relationship between the actual and predicted probabilities for the 1-, 3- and 5-year overall survival (OS).

### 2.5 Functional enrichment analysis

Kyoto Encyclopedia of Genes and Genomes (KEGG) pathway enrichment analysis was performed on differentially expressed genes (DEGs) by the R package “clusterProfiler” (v. 4.10.0) (Yu et al., 2012). DEGs were identified using the “DESeq2” algorithm (Love et al., 2014) between the high- and low-RILPS groups (p < 0.05, |log2foldchange| > 0.5). Additionally, a ridge plot and a correlation network diagram of signaling pathways were generated by “clusterProfiler” in R (Yu et al., 2012).

To explore the underlying molecular mechanisms of the RILPS model, single-sample gene set enrichment analysis (ssGSEA) of the gene expression profiles was used to identify the KEGG and Hallmark pathways predicted to be correlated with the RILPS score, with a p-value <0.05 considered statistically significant. The pathways of KEGG and Hallmark gene sets were downloaded from the MsigDB website (http://www.gseamsigdb.org/gsea/msigdb/collections.jsp) (Liberzon et al., 2011).

### 2.6 Correlation analysis

Pearson correlation analyses between the RILPS and clinical characteristics, including age, sex, race, P-stage, T-stage, M-stage, and N-stage were conducted and visualized via “ggplot” (v. 3.4.4) and “ggpubr” (v. 0.6.0) R packages. Similarly, correlation analyses between RILPS and immune cells, immune checkpoint genes, and mutated genes were also performed.

### 2.7 Immune cell infiltration

To explore the difference of immune cell infiltration between high- and low-RILPS groups, the infiltration of 28 immune cells was assessed using ssGSEA in the R “GSVA” package (v. 1.46.0) (Hänzelmann et al., 2013). Significant immune cells, including activated CD4 T cells, effector memory CD4 T cells, effector memory CD4 T cells, type 17 T helper cells, type 2 T helper cell, regulatory T cells, myeloid derived suppressor cells, NK T cells, activated dendritic cells, macrophages, eosinophils and monocytes were analyzed between low- and high-risk groups and visualized in box plots. Correlation coefficient was calculated to evaluate the relationship between immune cells and RILPS. The expression of immune checkpoints in low- and high-risk HCC groups was also analyzed. All immune-related signatures used in the tumor microenvironment analyses are shown in Supplementary Table S1, and the 28 immune cell signatures were obtained from Charoentong et al. (2017) (Charoentong et al., 2017).

### 2.8 Tumor mutational burden (TMB) and microsatellite instability (MSI) analysis

TMB was determined by calculating the mutation frequency and mutation/exon length of each sample, followed by dividing the non-synonymous mutation sites by the total length of the protein coding region (Wen et al., 2022). Clinical data regarding TMB and microsatellite instability (MSI) were obtained from the TCGA database.

### 2.9 Mutation analysis of RILPS

The mutation landscape was analyzed based on the cBioPortal (https://www.cbioportal.org/) to explore the mutation frequency and mutation spectrum of high- or low-RILPS HCC patients.

### 2.10 Drug sensitivity prediction

We used the “pRRophetic” package (v. 0.5) and the “oncoPredict” package (v. 1.2) to assess the drug sensitivity to several common drugs in high- and low-risk groups. The results were visualized using violin plots.

### 2.11 Patient recruitment

A total of 30 patients who underwent hepatic resection for HCC at the Department of Hepato-Biliary-Pancreatic Surgery, Ningbo No.2 Hospital between January 2025 and October 2025 were included for sample collection in this study. None of the patients had received preoperative treatments, including surgery, radiotherapy, chemotherapy, or biological/targeted therapies. Inclusion criteria were: a) HCC confirmed by morphological and histopathological examinations, with clinical manifestations, physical examinations, and auxiliary test results consistent with the diagnostic criteria for HCC; b) normal cardiac, pulmonary, and renal function, with no history of other malignancies or major systemic diseases; and c) complete clinicopathological data available, including sex, age, tumor size, degree of differentiation, lymph node metastasis, tumor stage, smoking and drinking history, family history of HCC, past medical history, and medication history. Patients were excluded if they met any of the following criteria: a) history of other malignancies; b) refusal or inability to provide informed consent; c) incomplete clinical data, poor compliance, or withdrawal during the study period.

Tumor and adjacent tissue samples were collected immediately after surgical resection, snap-frozen in liquid nitrogen, and stored at −80 °C until further analysis. This study was approved by the Ethics Committee of Ningbo No. 2 Hospital (PJ-NBEY-KY-2024-193-01). Written informed consent was obtained from all participants.

### 2.12 Reverse transcription-quantitative polymerase chain reaction (qPCR)

Total RNA was extracted from approximately 100 mg of sample tissue. Samples were fully ground in liquid nitrogen, followed by the addition of 1 mL TRIzol Reagent (15596026; Life Technologies/Thermo Fisher Scientific, Waltham, USA). RNA concentration and purity were measured using a nucleic acid protein analyzer (FC-3100, Life Real, Hangzhou, China). First-strand cDNA was synthesized using the FastKing RT Kit (with gDNase) (KR116, Tiangen, Beijing, China) according to the manufacturer’s instructions. Quantitative real-time PCR was performed using KAPA SYBR FAST qPCR Master Mix (2X) Universal (KK4601, Roche/KAPA Biosystems, Wilmington, USA). The qPCR amplification program was as follows: an initial denaturation at 95 °C for 15 s, followed by 40 cycles of 95 °C for 30 s and 60 °C for 60 s. Melting-curve analysis was conducted with a program of 95 °C for 60 s, 55 °C for 30 s, and 95 °C for 30 s to confirm amplification specificity. Target gene mRNA sequences were obtained from PubMed, and primers were designed based on the coding sequence (CDS) using Beacon Designer 7.90. All primers were synthesized by Invitrogen (Guangzhou, China). Relative gene expression levels were calculated using the 2^−^ΔΔCt method. Details of the primers used in this study are listed in Table 1.

**TABLE 1:** Primer sequences used for qPCR analysis.

Gene	Primer	Sequence (5′-3′)	Length (bp)	Amplicon size (bp)
RRM1	F	TAA​TTT​GGC​TTC​CCT​GGC​CC	20	169 bp
RRM1	R	GGG​GCG​ATG​GCG​TTT​ATT​TG	20	
RRM2	F	CCC​GCT​GTT​TCT​ATG​GCT​TC	20	181 bp
RRM2	R	TCT​TTG​TCC​CCA​ATC​CAG​CG	20	
GAPDH	F	GGA​CCT​GAC​CTG​CCG​TCT​AG	20	100 bp
GAPDH	R	GTA​GCC​CAG​GAT​GCC​CTT​GA	20	

### 2.13 Western blot (WB) analysis

Total protein was extracted from approximately 100 mg of frozen sample tissue using RIPA lysis buffer (Solarbio, Beijing, China) supplemented with 1 mM PMSF and phosphatase inhibitors (Solarbio, Beijing, China). Samples were homogenized on ice, incubated for complete lysis, and centrifuged at 12,000 rpm, 4 °C for 30 min to collect the supernatant. Protein concentrations were measured using a nucleic acid protein analyzer (FC-3100, Life Real, Hangzhou, China). Proteins were separated by 10% SDS-PAGE (Yamei, Shanghai, China) and transferred to PVDF membranes (IPVH00010; Millipore, Bedford, USA). Membranes were blocked with 5% non-fat milk in TBST at room temperature for 1.5 h, followed by incubation with primary antibodies at room temperature for 0.5 h and overnight at 4 °C. After washing, membranes were incubated with secondary antibodies for 1.5 h at room temperature, with three TBST washes between steps. Antibody details are provided in Table 2.

**TABLE 2:** Antibody information for WB analysis.

Antibody	Type	Brand	Cat. No.	Host	Dilution
RRM1 polyclonal	Primary	Proteintech	10526-1-AP	Rabbit	1:3,000
RRM2 polyclonal	Primary	Proteintech	11661-1-AP	Rabbit	1:3,000
GAPDH polyclonal	Primary	Proteintech	10494-1-AP	Rabbit	1:5,000
HRP-conjugated goat anti-rabbit IgG	Secondary	Proteintech	SA00001-2	Goat	1:5,000

### 2.14 Immunohistochemistry (IHC) analysis

Paraffin-embedded tissue sections were subjected to antigen retrieval in EDTA buffer (pH 9.0), followed by blocking of endogenous peroxidase activity with 3% hydrogen peroxide and nonspecific binding with 3% BSA. Sections were then incubated with primary antibodies overnight at 4 °C in a humidified chamber, followed by incubation with secondary antibodies at room temperature for 50 min. Color development was performed using DAB, and nuclei were counterstained with hematoxylin. Positive staining was indicated by brown or dark brown coloration, while nuclei appeared blue. Antibody details are provided in Table 3.

**TABLE 3:** Antibody information for IHC analysis.

Antibody	Type	Brand	Cat. No.	Host	Dilution
RRM1	Primary	Proteintech	10526-1-AP	Rabbit	1:200
RRM2	Primary	Proteintech	11661-1-AP	Rabbit	1:1,000
HRP-conjugated rabbit anti-goat IgG	Secondary	SeraCare	5,220–0362	Goat	1:100
HRP-conjugated goat anti-rabbit IgG	Secondary	SeraCare	5,220–0336	Goat	1:500
HRP-conjugated goat anti-mouse IgG	Secondary	SeraCare	5,220–0341	Goat	1:500
HRP-conjugated goat anti-rat IgG	Secondary	SeraCare	5,220–0364	Goat	1:500
HRP-conjugated goat Anti-Rabbit/Mouse (universal)	Secondary	DAKO	K5007	Goat	1:1

### 2.15 Cell culture and siRNA transfection

Human HCC cell line Huh7 was cultured in DMEM supplemented with 10% fetal bovine serum (FBS) at 37 °C in a humidified incubator containing 5% CO_2_. Three small interfering RNAs targeting LINC01138 (siRNA-1, siRNA-2, and siRNA-3) and a negative control (NC) siRNA were used for transient transfection. Cells were seeded into 6-well plates at a density of approximately 2 × 10^5^ cells/well and transfected when cell confluence reached 60%–70%.

siRNAs and transfection reagent were diluted in Opti-MEM and incubated at room temperature for 10–15 min to form transfection complexes, which were subsequently added to the cells. After 24 h, the culture medium was replaced with fresh complete medium. Forty-eight hours after transfection, total RNA was extracted and the knockdown efficiency of LINC01138 was evaluated by RT-qPCR. *GAPDH* was used as the reference gene.

### 2.16 CCK-8 cell proliferation assay

Cell proliferation was assessed using a Cell Counting Kit-8 (CCK-8; Beyotime, Shanghai, China). Briefly, 24 h after transfection, cells were collected and seeded into 96-well plates at a density of 3,000–5,000 cells per well. Cell viability was measured at 0, 24, 48, and 72 h. At each time point, 10 μL CCK-8 reagent was added to each well and incubated at 37 °C for 1–2 h. The absorbance at 450 nm was measured using a microplate reader.

### 2.17 EdU proliferation assay

Cell proliferation ability was further evaluated using an EdU Cell Proliferation Detection Kit (Beyotime, Shanghai, China). Forty-eight hours after transfection, cells were incubated with 10 μM EdU solution for 2 h, followed by fixation with 4% paraformaldehyde and permeabilization using 0.5% Triton X-100. Subsequently, Click reaction solution was added for fluorescent labeling, and nuclei were counterstained with DAPI. Images were captured under a fluorescence microscope, and the proportion of EdU-positive cells was quantified.

### 2.18 Wound healing assay

Cell migration ability was assessed using a wound healing assay. Transfected cells were seeded into 6-well plates and cultured to 90%–100% confluence. A sterile 200 μL pipette tip was used to create a linear scratch across the cell monolayer. Detached cells were removed by washing with PBS, and cells were subsequently cultured in serum-free or low-serum medium. Images of wound closure were captured at 0, 24, 48, and 72 h under an inverted microscope.

### 2.19 Statistical analysis

All statistical analyses were performed using R software (v. 4.3.0). Cox regression models were applied to assess the independent prognostic value of the RILPS. Student’s t-test was used to compare differences in immune cell infiltration, immune-related gene expression, and drug sensitivity between the two groups using the ggpubr package (v. 0.6.2). Spearman’s correlation analysis was used to explore relationships and calculate correlation coefficients between RILPS and clinical variables/frequently mutated genes. Kaplan-Meier (KM) survival curves were generated using the survminer package (v. 0.5.1). Log-rank tests were performed using the survival package (v. 3.8.3) to compare overall survival between high- and low-RILPS groups. Survival curves were visualized with ggplot2 (v. 3.5.2). Correlation analysis between RRM1/RRM2 and miRNAs was obtained from the Encyclopedia of RNA Interactomes (ENCORI) database (https://rnasysu.com/encori/panMirCoExp). Statistical significance was defined as p < 0.05.

## 3 Results

### 3.1 Increased RRM1 and RRM2 expression was associated with poor prognosis in HCC

**FIGURE 1:**
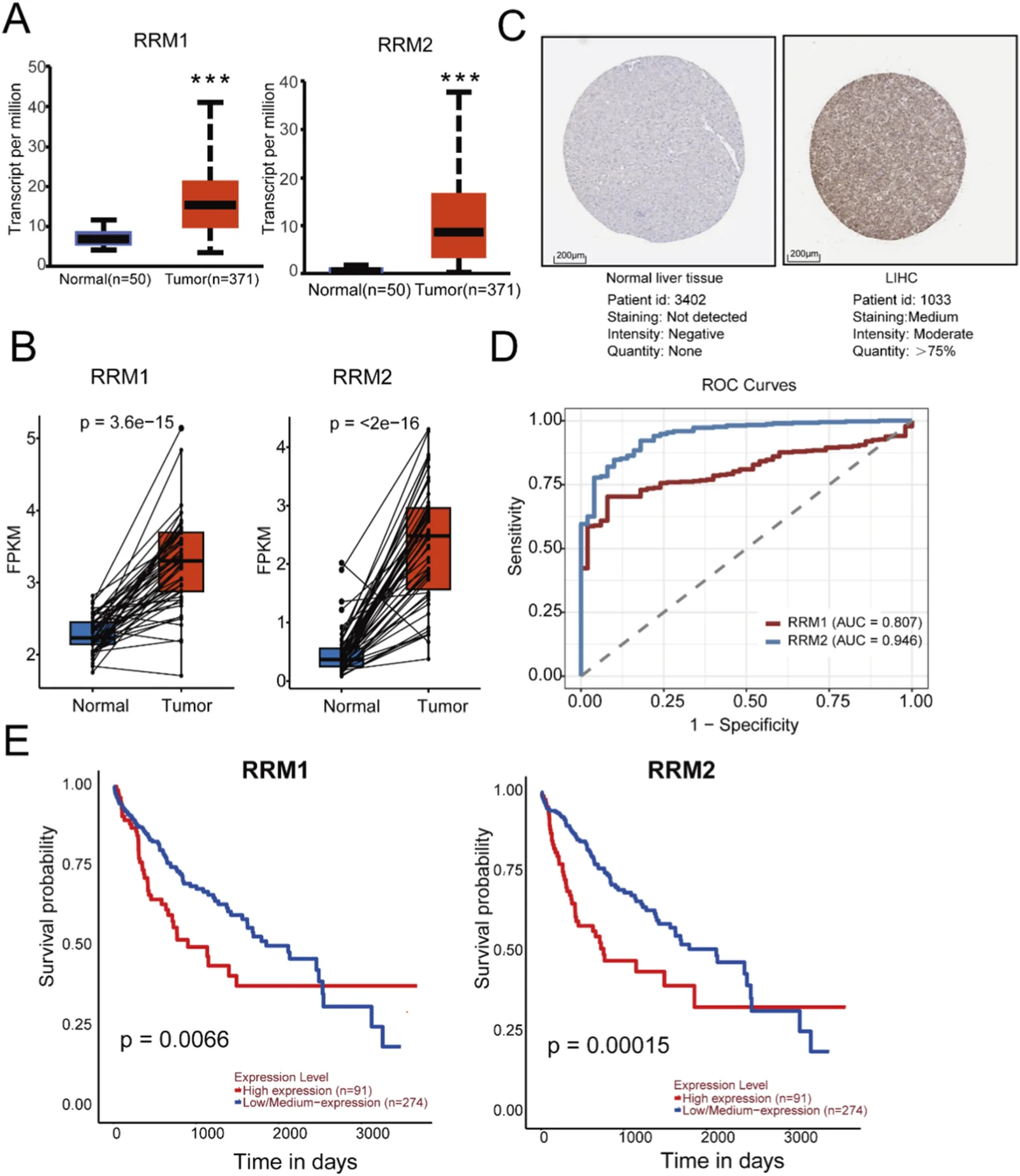
Expression of RRM1 and RRM2 and their association with prognosis in HCC. **(A)** RRM1 and RRM2 expression in both HCC and normal tissues in TCGA. **(B)** RRM1 and RRM2 expression in HCC and adjacent normal tissues in TCGA. **(C)** IHC images showing the expression of RRM1 and RRM2 in normal and HCC tissue obtained from the Human Protein Atlas (HPA) database. **(D)** ROC curves of RRM1 and RRM2 for distinguishing HCC from normal tissues. **(E)** Kaplan-Meier (KM) curves for the overall survival (OS) of HCC patients, stratified by high and low expression of RRM1/RRM2 in tumors.

To determine the association of RRM1/RRM2 expression with HCC patients, we compared their expression level in tumor tissues with that in normal controls from the TCGA dataset. The results showed that the mRNA expression of RRM1 and RRM2 was significantly increased in HCC tissues (p < 0.001; Figure 1A). Moreover, HCC tissues also exhibited higher expression of RRM1 and RRM2 than their paired adjacent non-tumor tissues (p < 0.001; Figure 1B). Immunohistochemical analyses were conducted to observe changes in protein levels of RRM1 and RRM2. Results of immunohistochemistry (IHC) images from the HPA database indicated that HCC tissues had significantly higher levels of RRM1 and RRM2 than normal liver tissues (Figure 1C). ROC analysis showed that RRM2 had excellent diagnostic value for HCC (AUC = 0.9456), while RRM1 showed moderate value (AUC = 0.8067) (Figure 1D). Finally, we investigated the correlation between RRM1/RRM2 expression and survival of HCC patients. KM curves indicated that higher expression levels of RRM1 and RRM2 were associated with poorer survival outcomes (Figure 1E). In conclusion, these results underscored the possible association between RRM1/RRM2 and HCC progression, as well as their negative impacts on patient survival, emphasizing the potential of these markers as prognostic indicators in HCC.

### 3.2 Delineating HCC subtypes through RRM1 and RRM2 expression profiling and LncRNA association analysis

**FIGURE 2:**
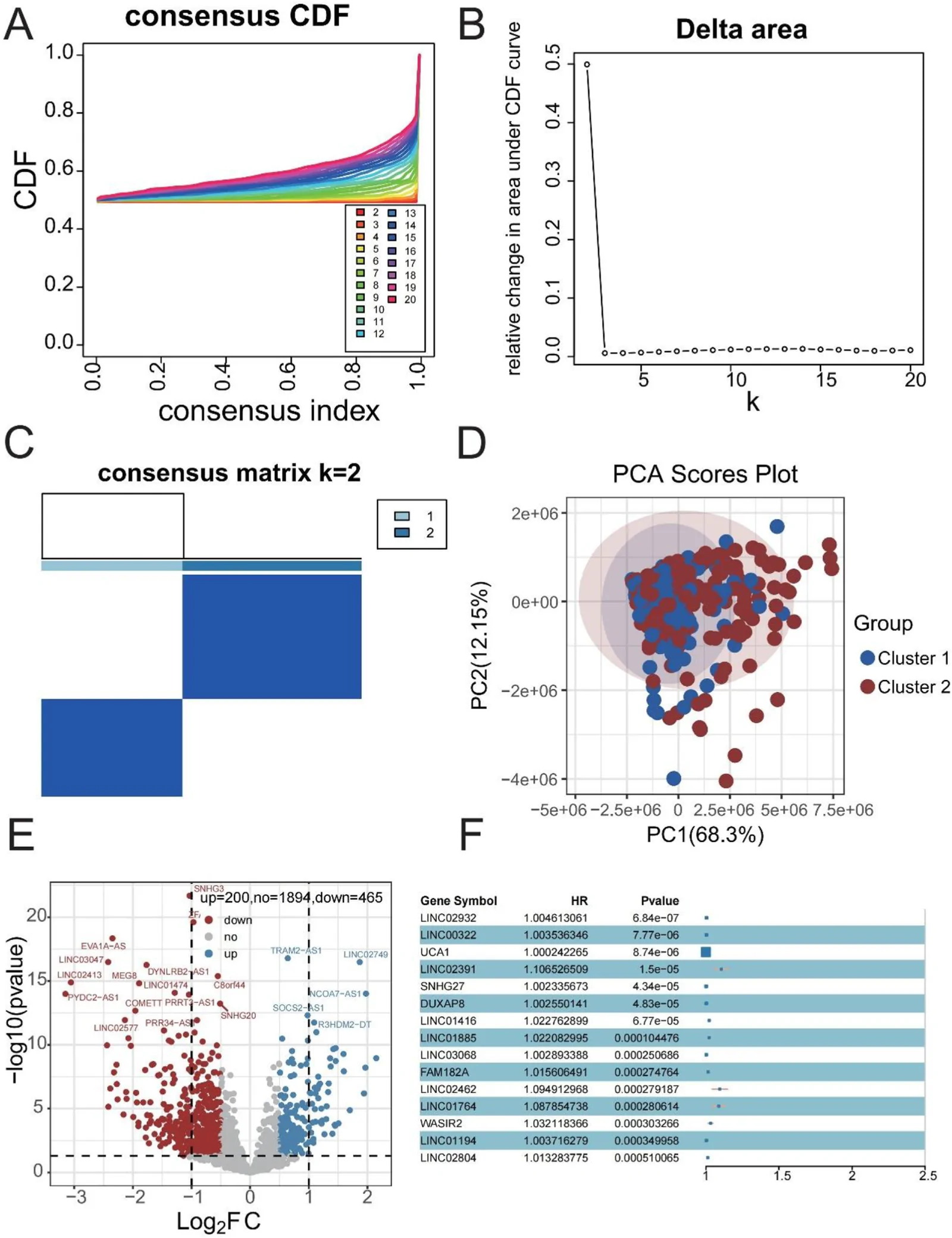
Exploration of RRM-associated LncRNAs. **(A)** Cumulative distribution function (CDF) curve of consensus clustering of RRM-related molecules (RRM1 and RRM2); the abscissa represents the consensus index, and the ordinate represents the CDF index. **(B)** Delta area plot for consensus clustering. The plot shows the relative change in the area under the CDF curve across different values of k, and the optimal k = 2. **(C)** Cluster heat map of RRM-associated molecular subtypes. **(D)** Principal component analysis (PCA) plot of RRM-related molecular subtypes. **(E)** Volcano plot showing the distribution of differentially expressed (DE)-LncRNAs. **(F)** Univariate cox regression analysis results of top 15 DE-LncRNAs. Only the top 15 lncRNAs ranked by statistical significance are displayed for visualization purposes, whereas all significant lncRNAs identified by univariate Cox regression were included in the subsequent analyses and model construction.

To gain insights into the effect of RRM1 and RRM2 on the HCC cohort, consensus clustering was performed to divide the TCGA samples into two groups based on their expression mode, namely, cluster 1 and cluster 2, according to the cumulative distribution function (CDF) (Figures 2A–C). Significant discrepancy was detected across these two clusters via principal component analysis (PCA) (Figure 2D). Next, we explored the functional difference between the two clusters. By using the R package “Deseq2”, a total of 1,406 DEGs between cluster 1 and cluster 2 were gathered (Supplementary Figure S1A). Further KEGG pathway analysis of DEGs exhibited the distinction of metabolism-related and cell cycle-related pathways (Supplementary Figure S1B), suggesting that RRM1 and RRM2 affecting HCC prognosis may be related to the activation of cell cycle-related pathways. LncRNAs have been reported to play important roles in cell proliferation, differentiation and development in animals and plants (Mattick et al., 2023). Therefore, we proceeded to perform a further analysis focusing on the differential expression of LncRNAs. A total of 665 DE-LncRNAs were detected, of which 200 were upregulated and 465 downregulated (Figure 2E). Investigation into the relationship between DE-LncRNAs and RRM1/RRM2 by Pearson correlation analysis revealed strong correlations (Supplementary Figure S1C), hinting that RRM1 and RRM2 may be regulated by these LncRNAs. This prompted us to further explore the possibility of DE-LncRNAs as prognosis factors in HCC patients. A univariate Cox regression analysis of these DE-LncRNAs was then performed, with 153 significant RRM-associated LncRNAs identified (Figure 2F) (Supplementary Table S2). Taken together, a strong association was identified between RRM1/2 and DE-LncRNAs, providing a foundation for the construction of prognostic models based on the expression profiles of the 153 LncRNAs.

### 3.3 Construction of an RRM-indexed LncRNA prognostic signature (RILPS) in stratifying risk and predicting survival in HCC

**FIGURE 3:**
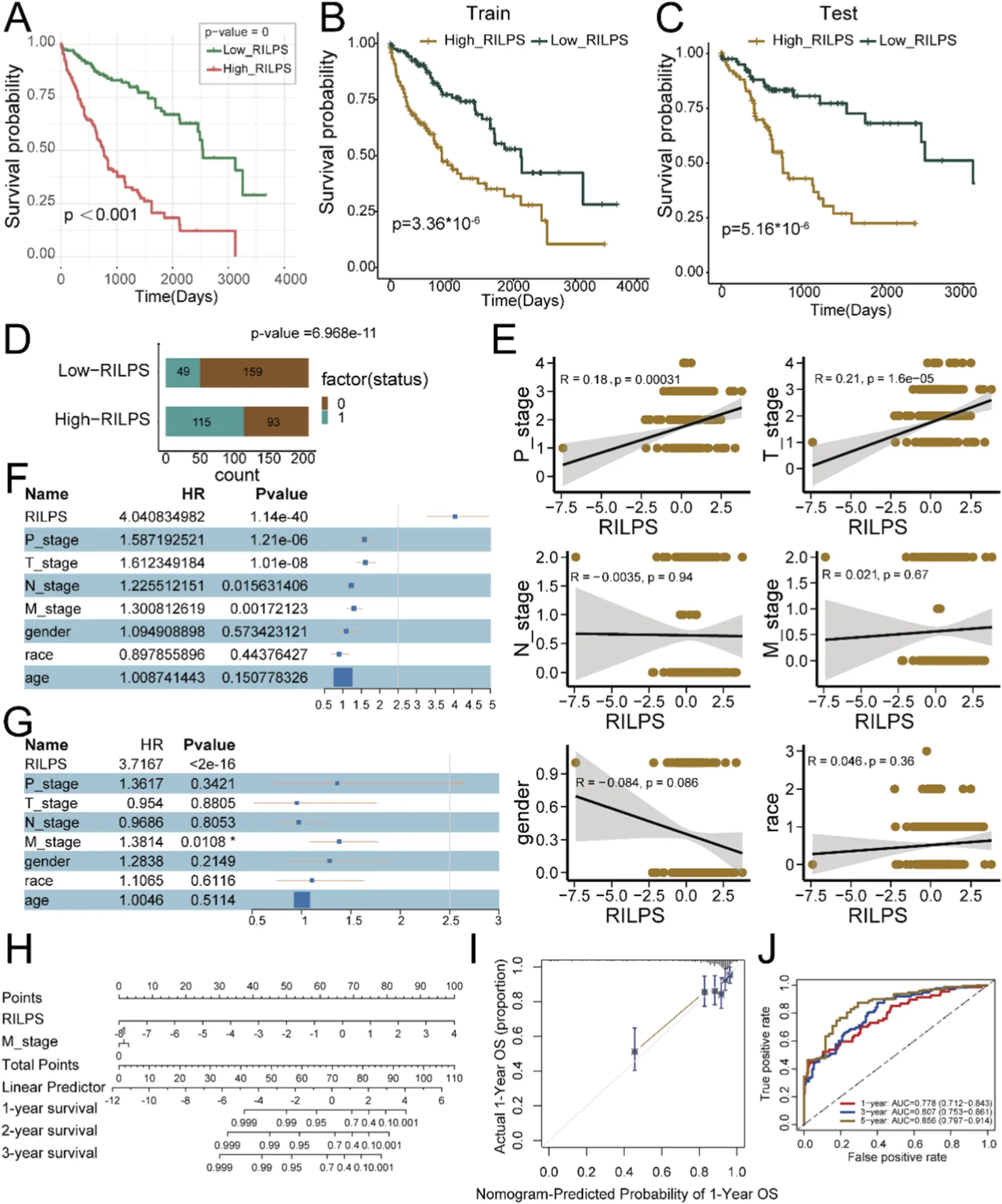
Construction of RILPS and its correlation with clinical characteristics. **(A)** Kaplan–Meier (KM) analysis for the overall survival (OS) of TCGA HCC cohort, as stratified by the RILPS Classifier. **(B)** KM analysis of TCGA Test cohort. **(C)** KM analysis of TCGA Training cohort. **(D)** Statistical results of the survival analysis of RILPS. **(E)** Correlation analysis between RILPS and clinical characteristics. **(F)** Univariate Cox regression analysis of RILPS and clinical characteristics. **(G)** Multivariate Cox regression analysis of RILPS and clinical characteristics. **(H)** Nomogram prediction model for HCC patients. **(I)** Calibration graph for the nomogram prediction model. The y-axis represents actual survival, and the x-axis represents nomogram-predicted survival. **(J)** The areas under the curve (AUCs) of the nomogram for 1-year, 3-year, and 5-year OS.

To construct a RILPS classifier using the 153 LncRNAs, the LASSO algorithm was applied, aiming to stratify HCC patients into two groups based on OS differences, namely, high- or low-RILPS groups. A total of 65 LncRNAs were selected for the construction of RILPS. KM survival curves were used to assess the prognostic value of the RILPS model. The results showed that the patients with high-RILPS had significantly reduced OS rate compared with those with low-RILPS across different data sets (Figures 3A–C). Additionally, a statistic analysis of OS indicated a higher number of deaths in the high-RILPS group (Figure 3D), which was consistent with the findings observed in the KM analysis. Subsequently, the relation between RILPS and clinical characteristics of HCC were investigated. Pearson correlation analysis indicted that the RILPS was significantly positively correlated with P- and T-stages, suggesting high-RILPS patients were characterized by a more advanced P-, T-stage and higher tumor grade (Figure 3E). However, after multivariable linear regression adjusting for P-, T-, N-, M-stage, gender, and race, these correlations were no longer significant (all p > 0.05, Supplementary Figure S1D), suggesting that the observed associations may be confounded by other clinical factors. Considering that the prognostically relevant clinical characteristics differed between the two risk groups, we further investigated whether RILPS had similar or better predictive validity with other HCC-independent prognostic factors. Therefore, we used univariate and multivariate logistic regression analyses to identify independent prognostic factors from RILPS, age, sex, race, T-, N-, and M-stage. In the univariate Cox regression analysis, RILPS, as well as P-, T-, N-, and M-stage, were significantly associated with OS (p < 0.05, Figure 3F), while in the multivariate Cox regression analysis, RILPS and M-stage emerged as independent predictors (HR > 1; Figure 3G). Finally, a predictive nomogram was constructed to assess the risk for each patient based on two independent prognostic factors, namely, RILPS and M-stage (Figure 3H). The score assigned to each factor was proportional to its risk contribution to survival (Figure 3H). The final nomogram scores for each patient, obtained by combining all factors, were used to predict 1-, 3-, and 5-year survival rates. The calibration curve demonstrated good agreement between the predicted and observed mortality (Figure 3I). The Hosmer–Lemeshow test further confirmed the good calibration of the nomogram (1-year: P = 0.318). The areas under the curve (AUCs) of the nomogram were 0.778, 0.807, and 0.856 for 1-year, 3-year, and 5-year OS, respectively (Figure 3J). To further validate the robustness of the RILPS model, we compared its prognostic performance with two well-established HCC protein biomarkers, GPC3 and AFP. Although GPC3 and AFP are clinically recognized protein-level prognostic markers, their mRNA expression levels alone showed limited predictive capacity in our transcriptomic cohort. In contrast, the RILPS model achieved significantly higher AUC values across all evaluated time points (1-, 3-, and 5-year), demonstrating superior prognostic accuracy and stability compared with single-gene models (Supplementary Figure S1E). These results may suggest that the RILPS can be used either as an independent prognostic factor or be integrated with existing clinical indicators.

Based on the above results, RILPS demonstrated excellent performance in stratifying HCC patients. To further elucidate the key LncRNAs contributing to the model, we sought to identify crucial LncRNAs within RILPS. Initially, the prognostic value of each signature LncRNA was assessed using Kaplan–Meier (KM) survival analysis. A total of 11 key LncRNAs significantly associated with HCC prognosis were identified (Supplementary Figure S2A). Specifically, the high expression of CT62, DLGAP1-AS3, KCNQ1-AS1, LINC00559, LINC01138, LINC01139, LINC02211, LINC01361, NBAT1, and WSIR2 was related to poor survival in HCC, whereas upregulated LINC00671 expression resulted in improved survival (Supplementary Figure S2A). Furthermore, Pearson correlation analysis revealed that most of these 11 lncRNAs were significantly positively correlated with RRM1 and RRM2 (Supplementary Figure S2B). Combining the prognostic significance and expression patterns of these LncRNAs in the risk score model grouping (Supplementary Figure S2C), three LncRNAs (NBAT1, LINC01138, and LINC00671) were identified as key regulators in HCC. Notably, NBAT1 and LINC01138 were upregulated in the high-RILPS group, whereas LINC00671 was upregulated in the low-RILPS group. Further ROC curve analysis showed that LINC01138 exhibited strong diagnostic performance in distinguishing HCC tissues from normal tissues (AUC = 0.922) (Supplementary Figure S2D).

Next, we checked the correlation between the three LncRNAs and cell cycle-related or cell metastasis-related gene sets. The results showed that NBAT1 and LINC01138 was significantly positively correlated with cell cycle-related and cell migration-related gene sets (Supplementary Figure S3A), while LINC00671 showed a negative correlation with them (Supplementary Figure S3C). Thus, the upregulation of NBAT1 and LINC01138, along with the downregulation of LINC00671, may contribute to the progression of HCC by promoting tumor cell proliferation and migration.

### 3.4 Low-RILPS HCC was linked to activated metabolism-related pathways

**FIGURE 4:**
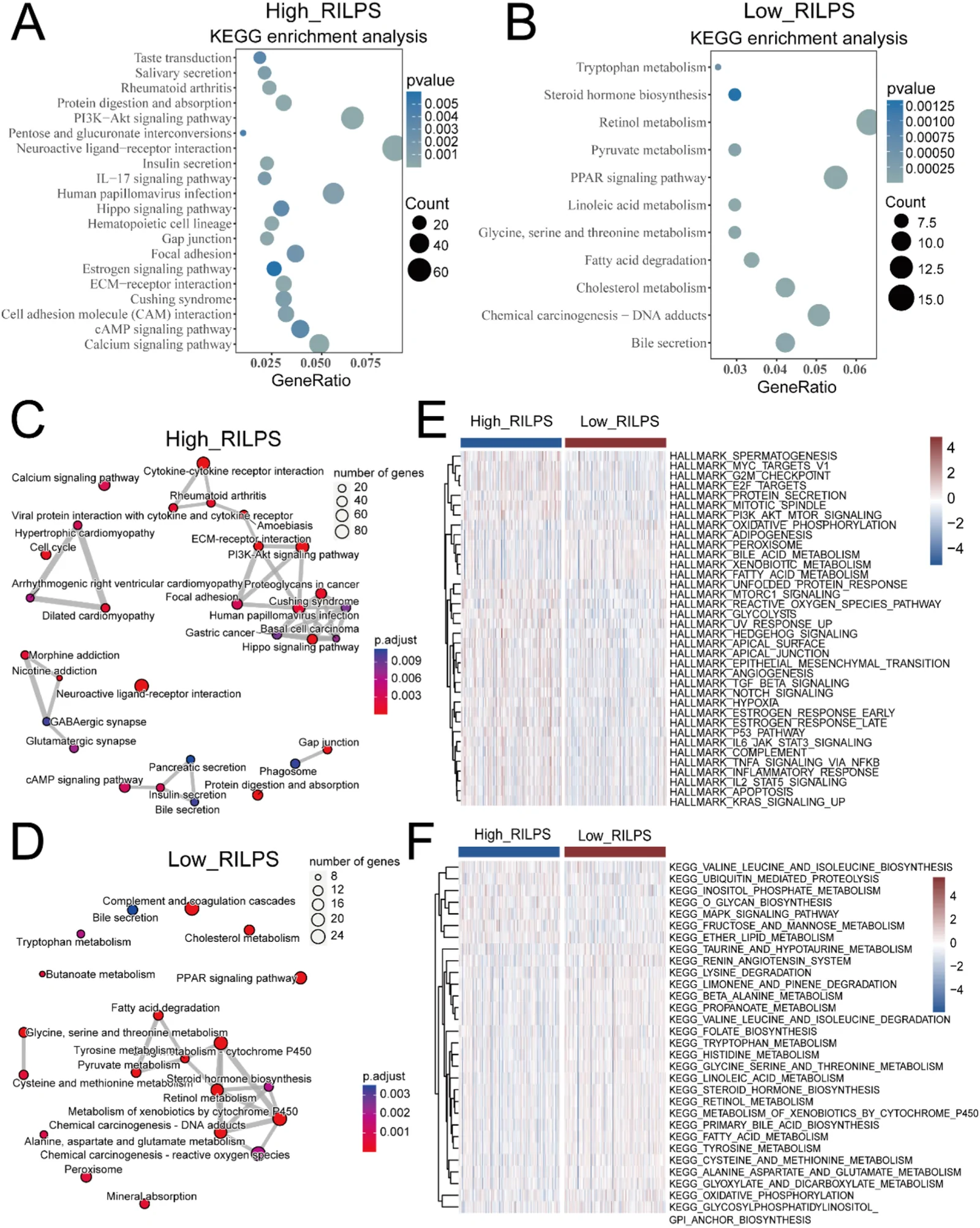
Gene function enrichment analyses. **(A,B)** KEGG enrichment analysis of upregulated DEGs in the high-RILPS group **(A)** and low-RILPS group **(B)**. Pathway interaction network of DEGs in high-RILPS **(C)** and low-RILPS **(D)** groups **(E)**. Heatmap showing the expression levels of Hallmark gene sets obtained via ssGSEA **(F)**. Heatmap showing the expression levels of KEGG gene sets obtained via ssGSEA.

To investigate differences in biological functions and pathways between the high-and low-RILPS groups, KEGG enrichment analysis was performed. The genes upregulated in high-RILPS group were mainly associated with “PI3K-Akt signaling pathway”, “IL-17 signaling pathway”, “focal adhesion”, and “extracellular matrix (ECM)-receptor interaction” (Figure 4A). Notably, both the PI3K-Akt and IL-17 signaling pathways are known to promote the proliferation and survival of tumor cells, as well as drug resistance, thereby contributing to unfavorable prognostic outcomes. In contrast, various metabolism-related pathways, including “tryptophan metabolism”, “steroid hormone biosynthesis”, “retinol metabolism”, “linoleic acid metabolism”, “fatty acid degradation” and “cholesterol metabolism” were enriched in low-RILPS patients (Figure 4B). Additionally, the pathway interaction network exhibited relationships among these pathways in high- or low-RILPS groups (Figures 4C,D). In the high-RILPS group, a strong relationship was observed among “ECM-receptor interaction”, “PI3K-Akt signaling pathway”, “cell cycle”, “focal adhesion”, and “hippo signaling pathway” (Figure 4C). For the low-RILPS population, a distinct interaction network involving several metabolic signaling pathways was identified (Figure 4D). Thereafter, ssGSEA based on Hallmark and KEGG gene sets was performed to detect differences between the two groups. The results of the Hallmark gene sets indicated differences in tumor-related, and energy metabolism-related pathways, which were mainly activated in the high-RILPS group (Figure 4E). Furthermore, “fatty acid metabolism”, “primary bile acid biosynthesis”, “steroid hormone biosynthesis”, “linoleic acid metabolism” “folate biosynthesis” and “valine leucine and isoleucine degradation” were significantly enhanced in the low-RILPS group regarding KEGG gene sets (Figure 4F). Our findings thus identified active metabolism as a key feature for low-RILPS patients, potentially facilitating nutrient supply for tumor cell growth and proliferation.

### 3.5 High-RILPS HCC was characterized by enhanced immune infiltration and checkpoint expression

**FIGURE 5:**
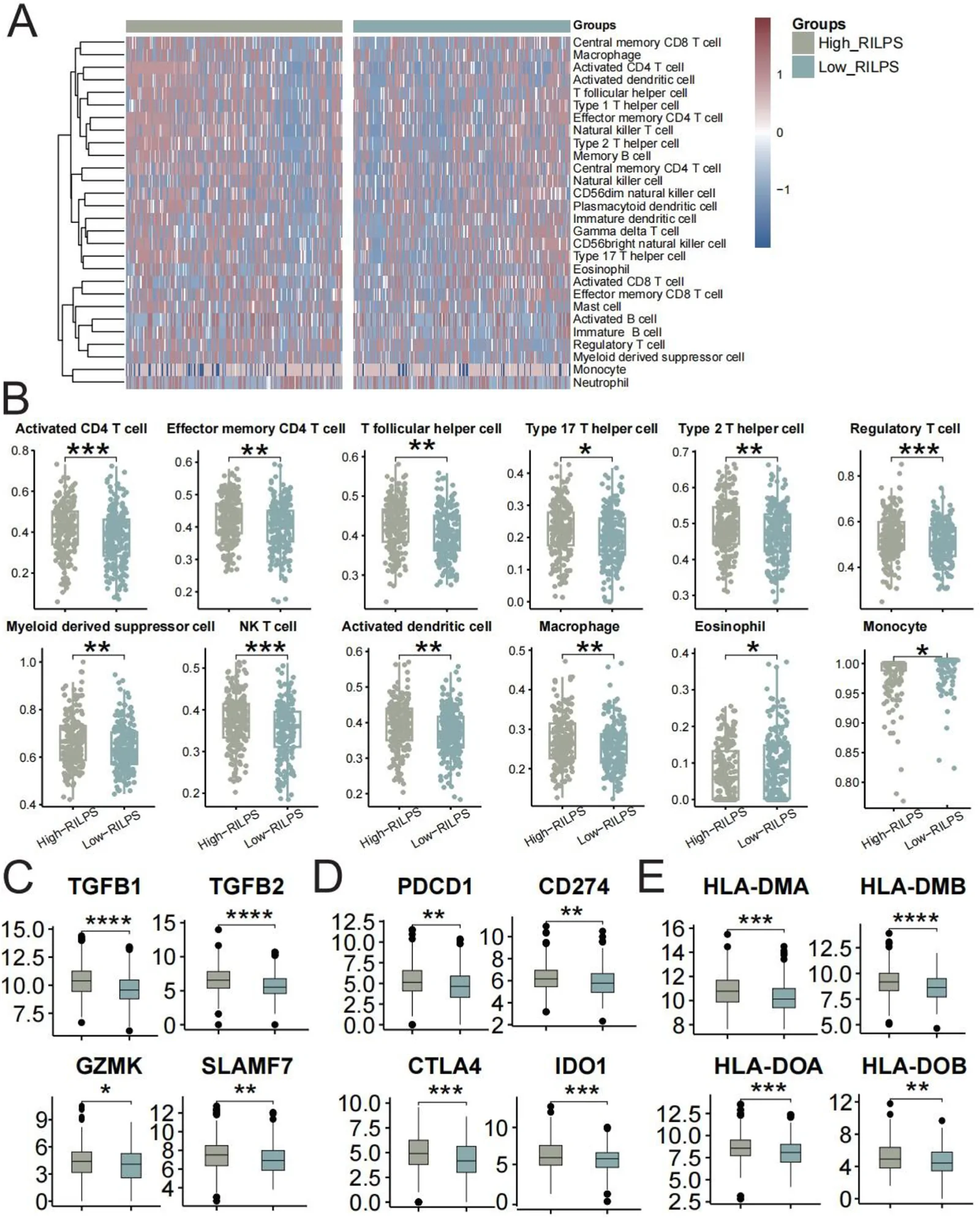
Immune infiltration analysis of RILPS. **(A)** Immune profiles between high- and low-RILPS patients, showing enrichment levels of 28 tumor microenvironment (TME)-related immune cells. **(B)** Significant differences in immune cell infiltration between high- and low-RILPS groups (t-test, p ≤ 0.05). **(C)** Differences in the expression levels of immune biomarkers between high- and low-RILPS patients. **(D)** Differences in the expression levels of immune checkpoint genes between high- and low-RILPS patients. **(E)** Differences in the expression levels of human leukocyte antigen (HLA) genes between high- and low- RILPS patients. * indicates p < 0.05; ** indicates p < 0.01; *** indicates p < 0.001.

To further elucidate changes in the immune microenvironment in HCC patients, ssGSEA was used to estimate the extent of 28 immune cell infiltration (Figure 5A). Specifically, high-RILPS patients exhibited higher immunocyte infiltration levels of activated CD4 T cell, effector memory CD4 T cell, type 17 T helper cell, type 2 T helper cell, regulatory T cell, myeloid derived suppressor cell, NK T cell, activated dendritic cell, macrophage, *etc.* (p < 0.05; Figure 5B). Correlation analysis further confirmed their significant positive correlation with RILPS (Supplementary Figure S4A). Besides, we also investigated expression differences in immune-related, immune checkpoint-related, HLA-related genes between the two groups. As shown in the box plots, the expression of these genes in the high-RILPS group was significantly higher than that in the low-RILPS group (Figures 5C–E). The correlation results indicated a positive correlation between the expression of immune-related genes and RILPS (Supplementary Figure S4B). In summary, high-RILPS patients were characterized by enhanced immune infiltration and checkpoint expression.

### 3.6 Distinct mutation profiles and gene expression patterns defined risk stratification in HCC

**FIGURE 6:**
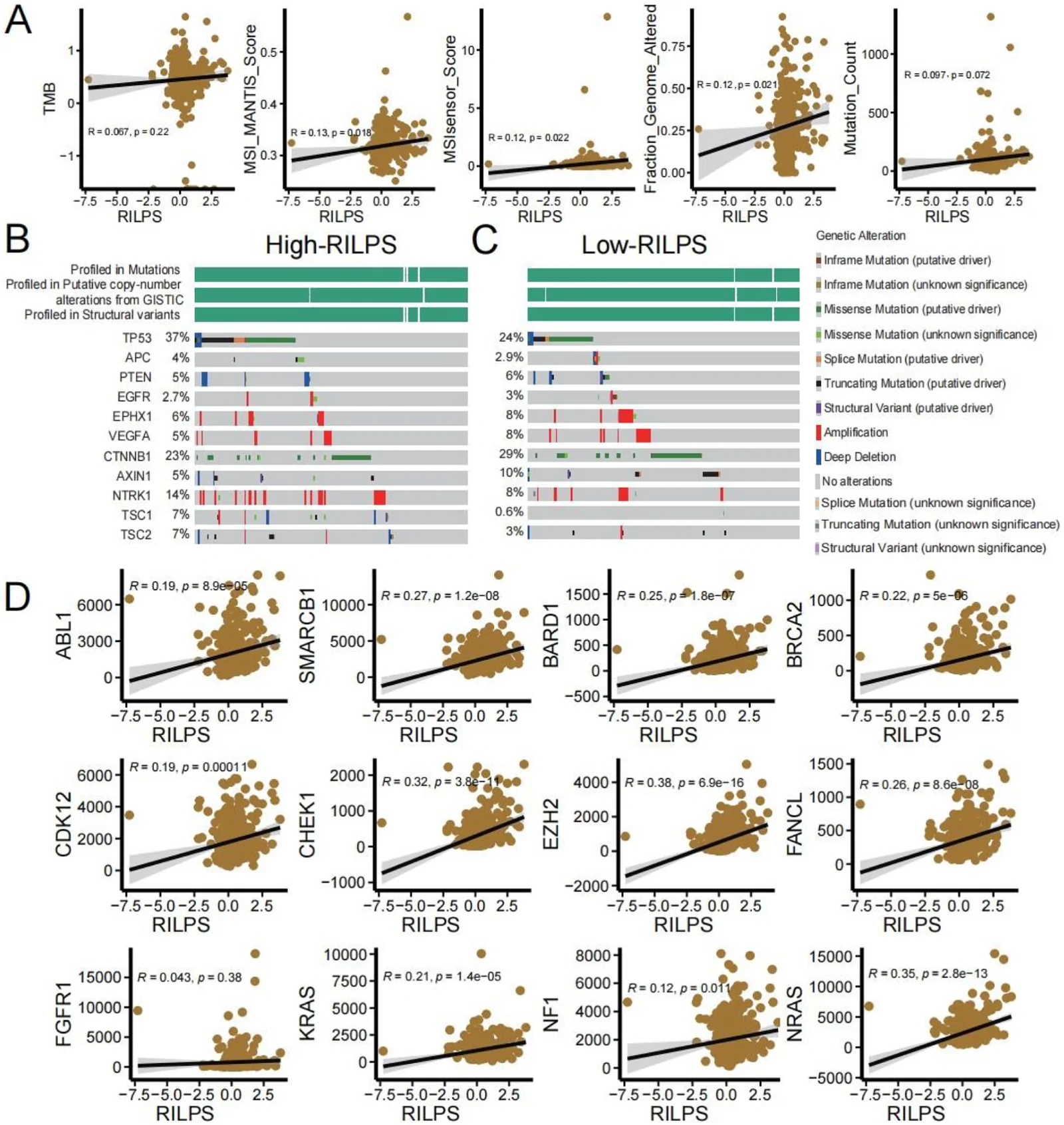
Mutation features of high- and low-RILPS HCC patients **(A)**. Correlation between TMB/MSA/FGA/mutation count and RILPS. **(B,C)**. Mutation landscape of high-RILPS **(B)** and low-RILPS groups **(C)**. **(D)** Correlation analysis between protooncogenes and RILPS.

Currently, MSI and TMB are important prognostic indicators for patients with tumors and can guide treatment decisions, especially regarding the use of immune checkpoint inhibitors (ICIs) (DeStefanis et al., 2019). Therefore, we estimated the relationship between MSI, TMB, fraction of genome altered (FGA), and RILPS in HCC. Our results revealed that RILPS was positively associated with MSI, TMB, and FGA, suggesting that patients with high-RILPS scores may benefit from ICIs (Figure 6A). Additionally, we further explored the relationship between RILPS and mutations in HCC. The mutation profile of most frequently mutated genes in HCC in the high- and low-RILPS groups is demonstrated in Figures 6B,C. The five mutated genes, namely TP53, APC, NTRK1, TSC1 and TSC2, exhibited a higher frequency of mutations and a more diverse mutation spectrum in the high-RILPS group (Figure 6B). In contrast, PTEN, EGFR, EPHX1, VEGFA, CTNNB1, and AXIN1 were more frequently mutated in the low-RILPS group (Figure 6C). Besides, we investigated the association between RILPS and several common protooncogenes. The results revealed a significantly positive correlation (Figure 6D). In general, the high-RILPS group was more likely to respond to immunotherapy because of higher levels of MSI, TMB, and FGA.

### 3.7 Screening of potential therapeutic drugs for HCC patients

**FIGURE 7:**
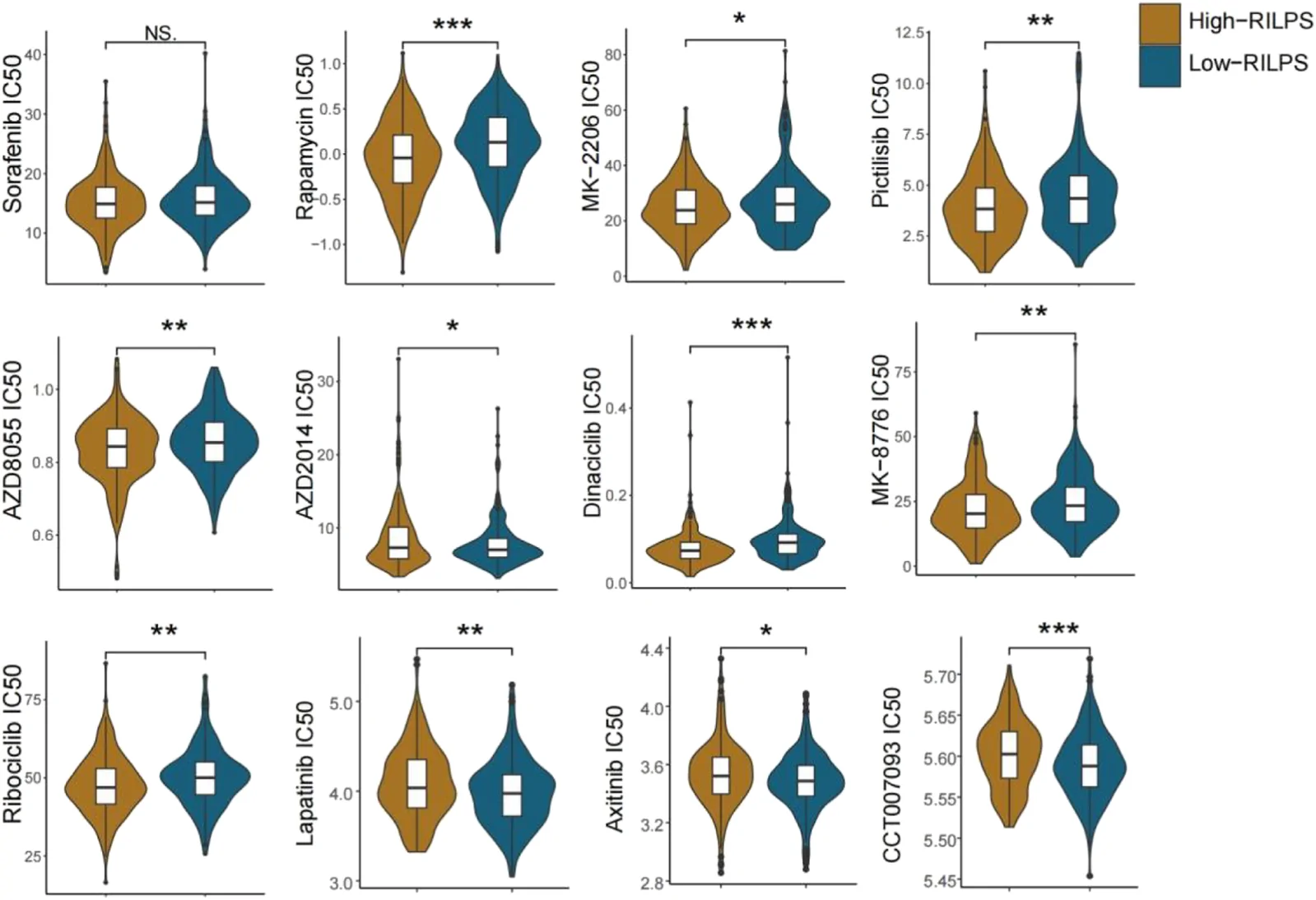
Drug sensitivity in high- and low-RILPS HCC patients. * indicates p < 0.05; ** indicates p < 0.01; *** indicates p < 0.001.

Based on functional enrichment analysis, the RILPS signature was found to be associated with tumor proliferation and survival pathways, which represent key druggable pathways in HCC. We therefore performed drug sensitivity analysis using the pRRophetic and oncoPredict packages. Sorafenib, the standard first-line therapeutic agent for HCC, was included as a control to evaluate the clinical relevance of the RILPS signature. Representative drugs targeting pathways dysregulated in high-RILPS patients were selected, including PI3K/MTOR inhibitors (rapamycin, MK-2206, pictilisib, AZD8055, AZD 2014), cell cycle regulators (dinaciclib, MK-8776, ribociclib), and small molecule enzyme inhibitors (lapatinib, axitinib, CCT007093). The half-maximal inhibitory concentration (IC50) values were then compared between the high- and low-RILPS groups. Analysis of these 12 agents revealed distinct patterns of drug sensitivity between patient subgroups. Specifically, lapatinib, axitinib, and CCT007093 exhibited significantly lower IC50 values in low-RILPS HCC patients, indicating higher sensitivity to these drugs in this subgroup. In contrast, high-RILPS patients demonstrated greater sensitivity, as indicated by lower IC50 values, to drugs targeting the PI3K/MTOR pathway and the cell cycle, including rapamycin, MK-2206, pictilisib, AZD8055, AZD 2014, dinaciclib, MK-8776, and ribociclib (Figure 7). These findings underscore the importance of RILPS features in HCC treatment, as they can guide clinicians in selecting the most effective therapeutic agents based on the individual patient’s tumor profile. By leveraging the IC50 data, we can enhance therapeutic efficacy, ultimately improving patient outcomes.

### 3.8 Experimental validation of RRM1 and RRM2 expression in HCC tissues

**FIGURE 8:**
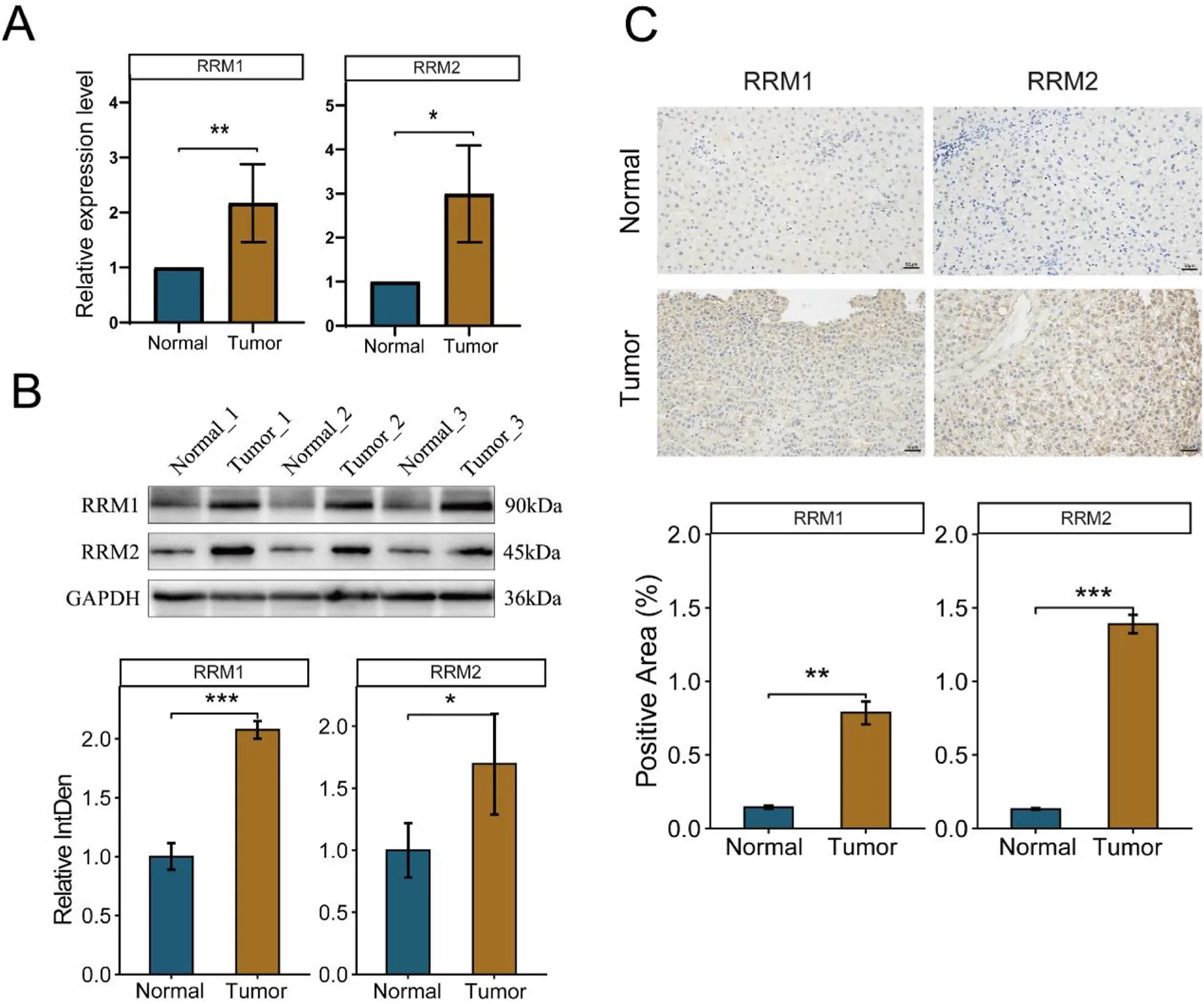
Experimental validation of RRM1 and RRM2 expression in HCC tissues. **(A)** qPCR analysis of RRM1 and RRM2 expression in paired HCC tissues and adjacent non-tumor tissues. Data are presented as mean ± SD; statistical significance was determined using paired t-tests. *p < 0.05, **p < 0.01. **(B)** Western blot analysis showing increased protein levels of RRM1 and RRM2 in paired HCC and adjacent non-tumor tissues. **(C)** Representative immunohistochemical (IHC) staining confirmed significantly higher RRM1 and RRM2 expression in tumor tissues compared with adjacent controls.

To experimentally validate the bioinformatic predictions, we examined the expression of RRM1 and RRM2 in paired HCC and adjacent non-tumor tissues using qPCR, Western blotting, and immunohistochemistry. qPCR analysis revealed that both RRM1 and RRM2 were significantly upregulated in tumor tissues compared with matched non-tumor controls (Figure 8A). Consistently, WB confirmed markedly elevated protein levels of RRM1 and RRM2 in HCC samples (Figure 8B). Immunohistochemical staining further demonstrated strong positive staining of RRM1 and RRM2 in tumor regions, whereas adjacent liver tissues exhibited weak or minimal signals (Figure 8C). Collectively, these results support the role of RRM1 and RRM2 as key components of the prognostic signature.

### 3.9 Effect of LINC01138 on proliferation and migration of HCC cells

**FIGURE 9:**
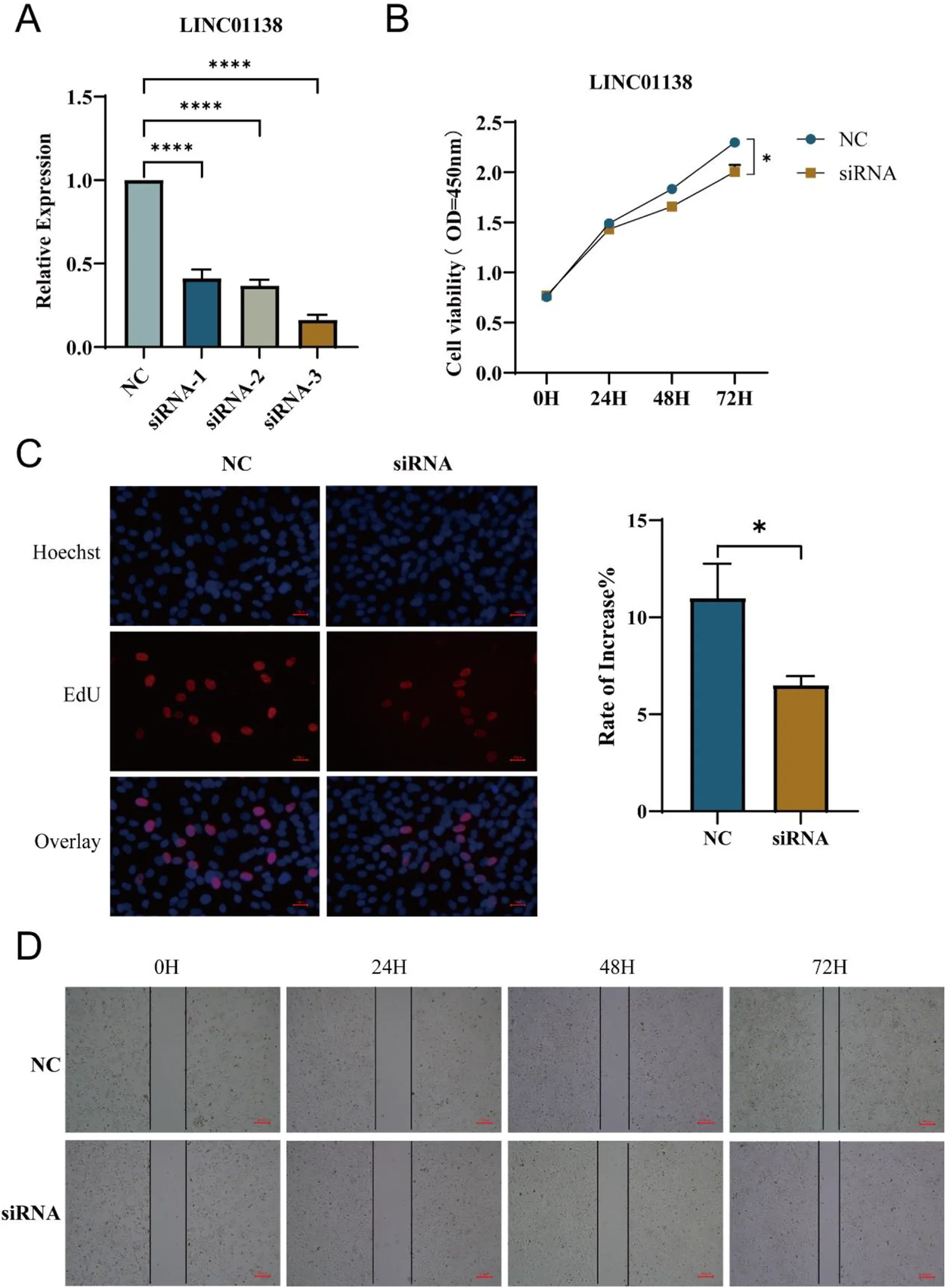
Effects of LINC01138 knockdown on proliferation and migration of HCC cells. **(A)** qPCR detection of LINC01138 expression levels in Huh7 cells after transfection with three small interfering RNAs targeting LINC01138 (siRNA1, siRNA2, siRNA3) or negative control (NC). **(B)** CCK-8 assay assessing the proliferative activity of Huh7 cells at different time points (0, 24, 48, 72 h) following LINC01138 knockdown. **(C)** EdU staining assay detecting the proportion of EdU-positive Huh7 cells after LINC01138 knockdown (scale bar = 20 μm). **(D)** Cell scratch (wound healing) assay examining the scratch closure of Huh7 cells at 0, 24, 48, and 72 h following LINC01138 knockdown (scale bar = 200 μm).

Given that bioinformatics analysis indicated an association between LINC01138 and poor prognosis in HCC (Supplementary Figure S2), we further conducted *in vitro* functional experiments to preliminarily investigate its impact on the biological behavior of HCC cells. We designed three small interfering RNAs (siRNA1, siRNA2, siRNA3) targeting LINC01138 and transfected them into Huh7 cells. qPCR results showed that, compared with the negative control (NC) group, all three siRNAs significantly reduced the expression level of LINC01138, with siRNA3 exhibiting the highest knockdown efficiency (Figure 9A). CCK-8 assay results demonstrated that following LINC01138 knockdown, the proliferative capacity of Huh7 cells significantly decreased over time (Figure 9B). Consistently, EdU staining assays revealed a markedly lower proportion of EdU-positive cells in the LINC01138 knockdown group than in the NC group (Figure 9C), further confirming the inhibitory effect of LINC01138 silencing on HCC cell proliferation. In addition, wound healing assays demonstrated that the wound closure area was markedly reduced in the LINC01138 knockdown group at 24, 48, and 72 h compared with the NC group (Figure 9D), indicating that LINC01138 silencing impaired the migratory capacity of Huh7 cells. In summary, these *in vitro* experimental results indicate that high expression of LINC01138 promotes proliferation and migration of HCC cells. These experimental results provide preliminary biological evidence supporting the prognostic relevance of LINC01138 identified in our multi-lncRNA prognostic model, although further mechanistic studies are still required.

## 4 Discussion

As a key rate-limiting enzyme in DNA synthesis and repair, RR performs significant roles in a variety of bioprocesses, such as cell proliferation and differentiation (Aye et al., 2014). Recent studies have reported RRMs as independent risk factors for the prognosis of patients with pancreatic cancer (Han et al., 2018), non-small cell lung cancer (Zhu et al., 2018) and breast cancer (Abdel-Rahman et al., 2022). Therefore, LncRNAs could be underlying molecular target for RRMs (Gandhi et al., 2020). However, the regulatory mechanisms governing RRMs and their interplay with LncRNAs in HCC remain elusive. It is thereby imperative to explore the interaction between RRMs and LncRNAs to identify effective prognostic markers for HCC. In this study, we found that the expression patterns of RRM1 and RRM2 were associated with specific lncRNAs, leading to the identification of numerous DE-lncRNAs. Based on these RR-related lncRNAs, we constructed a prognostic model consisting of 65 lncRNAs (Supplementary Table S3 **)**. Among them, several representative lncRNAs have been previously reported to participate in tumor progression and cancer-related biological processes. NBAT1 is generally regarded as a tumor-suppressive lncRNA in various cancers, where it inhibits tumor cell proliferation, migration, and invasion and is associated with favorable clinical outcomes (Yu et al., 2024; Yan et al., 2017; Sethi et al., 2025). In contrast, LINC01138 has been widely reported as an oncogenic lncRNA that promotes HCC progression through regulation of tumor cell proliferation and oncogenic signaling pathways (Li et al., 2018; Xiao et al., 2022; Dou et al., 2019). Similarly, LINC00671 has been reported to exert tumor-suppressive effects in multiple cancers by inhibiting tumor cell proliferation and metastasis through regulation of oncogenic signaling pathways such as AKT/ERK signaling, miRNA-mediated regulatory networks, and glycolysis-related pathways (Huo et al., 2021; Zhang et al., 2018; Qu et al., 2021a; Jin et al., 2021). These findings indicate that RILPS-related lncRNAs may be closely involved in cancer-associated biological processes. However, it should be noted that the RILPS signature was established based on the combined prognostic contribution of multiple lncRNAs rather than the independent biological effect of a single molecule. Therefore, the prognostic performance of the signature reflects the integrated effect of the overall lncRNA expression pattern rather than strictly corresponding to the individual functional characteristics reported for each lncRNA in previous studies. This may be attributed to the fact that lncRNAs often exert context-dependent regulatory functions in HCC, where their biological roles are influenced by tumor heterogeneity, microenvironmental cues, and interaction networks such as competing endogenous RNA (ceRNA) systems (Liu et al., 2018; Yang et al., 2023; Xu et al., 2020). Under such conditions, the same lncRNA may participate in different regulatory axes, and its net effect on tumor progression is determined by the dominant signaling pathways and molecular partners within a given pathological context. The high- and low-RILPS groups exhibited distinct pathway enrichment profiles, with the high-risk group enriched in pathways related to cell proliferation and drug resistance, while the low-risk group showed enrichment in metabolism-related pathways. RRM1 and RRM2 may influence the activation of these pathways through the regulation of lncRNAs. Therefore, our findings suggest that RRM-related lncRNAs may participate in HCC progression and are associated with biological pathways involved in proliferation, drug resistance, and metabolism. These associations provide a basis for future mechanistic studies to clarify their functional roles in HCC.

Based on the prognostic difference between high-RILPS and low-RILPS groups, we performed functional enrichment analyses of KEGG and ssGSEA in DEGs in these groups. The high-RILPS group was primarily associated with the “PI3K-Akt signaling pathway,” “IL-17 signaling pathway,” “focal adhesion,” and “ECM-receptor interaction.” The activation of these pathways can promote the proliferation and migration of tumor cells. Conversely, low-RILPS patients were mainly enriched in metabolism-related pathways, including “tryptophan metabolism”, “steroid hormone biosynthesis”, “retinol metabolism”, “linoleic acid metabolism”, “fatty acid degradation” and “cholesterol metabolism”. ssGSEA results also associated low-RILPS patients with activated metabolism-related pathways, further supporting these findings.

The type and quantity of infiltrating immune cells in the tumor microenvironment are closely related to the development of the tumor, and affect the strategy and effectiveness of immunotherapy. The immune system can exert both anti-tumor and pro-tumor activities. Therefore, we further explored discrepancies between the two groups. Via ssGSEA of 28 immune cells, we discovered that the high-RILPS group was characterized by high levels of seven kinds of immune cells (activated CD4 T cell, activated dendritic cell, effector memory CD4 T cell, regulatory T cell, T follicular helper cell, macrophage, and natural killer T cell), suggesting that HCC with high-RILPS scores may be more sensitive to immunotherapy. However, the higher degree of immune infiltration in the high-RILPS group was paradoxically associated with poorer overall survival. This finding may reflect an immunosuppressive tumor microenvironment characterized by immune remodeling and immune exhaustion (Shen et al., 2024; Yin et al., 2024), rather than an effective anti-tumor immune response (Zhao J. et al., 2025; Yan et al., 2018). The enrichment of immunosuppressive cell populations, such as regulatory T cells (Tregs), may further contribute to impaired immune surveillance and unfavorable clinical outcomes. Therefore, increased immune infiltration should not necessarily be interpreted as enhanced anti-tumor immunity but may instead indicate a functionally suppressed immune state in high-RILPS tumors (Yuan et al., 2023; Hu et al., 2025). Nevertheless, these interpretations remain speculative and require further experimental validation.

The differential mutation patterns observed between the high- and low-RILPS groups may have important clinical implications. In the high-RILPS group, TP53 mutations were more frequently observed. As a well-established tumor suppressor gene, mutations in TP53 have been associated with increased tumor aggressiveness, poor differentiation, and unfavorable prognosis (Joerger et al., 2025; Zhao K. et al., 2025). APC mutations may contribute to tumor progression through dysregulation of the Wnt/β-catenin signaling pathway, a common consequence of APC loss-of-function in HCC (Lehrich and Monga, 2026; Wu et al., 2025). In addition, enrichment of mutations in NTRK1, TSC1, and TSC2 was also observed in this group. Notably, TSC1/2 alterations are known to activate the mTOR signaling pathway, which has been implicated in tumor progression (Zhao N. et al., 2025; Inoki et al., 2003). Collectively, these findings suggest that the high-RILPS group may be characterized by a more aggressive molecular phenotype. These results further highlight the potential utility of RILPS in stratifying HCC patients with distinct genomic and biological characteristics, which may be valuable for prognosis assessment and personalized therapeutic decision-making, although further validation in independent cohorts is warranted.

Thereafter, we examined the independent prognostic value of 65 LncRNAs in RILPS, 11 of which were significant. In consideration of their expression, three LncRNAs (NBAT1, LINC01138, and LINC00671) were identified to be crucial in HCC. Previous studies have found that NBAT1 inhibits the progression of renal carcinoma (Xue et al., 2019) and breast cancer (Hu et al., 2015), while the NBAT1 expression has been reported to be decreased in HCC tissues and cells (Wei et al., 2021). These findings indicate the role of NBAT1 as a tumor suppressor, which is inconsistent with our results. We speculate that this inconsistency may be caused by the diversity of HCC samples and the heterogeneity of HCC. Moreover, Wei et al. analyzed HCC tissues from a single hospital, potentially limiting the representativeness of their study. LINC01138 may promote cell proliferation, tumorigenicity, tumor invasion and metastasis by activating arginine methyltransferase 5 in HCC (Li et al., 2018). Silencing LINC01138 can inhibit the glycolysis of glioma cells by regulating the microrNA-375/SP1 axis, thereby reducing proliferation (Xu et al., 2021). Similarly, Dou et al. (Dou et al., 2019) found that LINC01138 activated the MAPK signaling pathway by inhibiting miR-1273e, this activation may promote the proliferation, invasion and migration of gastric cancer cells, while inhibit the apoptosis of these cells. These results confirm the carcinogenic effect of LINC01138 and are consistent with our findings. Importantly, we found for the first time that LINC00671 was downregulated in HCC and negatively correlated with cell cycle-related and cell migration-related gene sets. In fact, LINC00671 has been reported to be downregulated in a variety of tumors, such as pancreatic cancer (Zhang et al., 2018), renal cell carcinoma (RCC) (Jin et al., 2021), and papillary thyroid tumor (Huo et al., 2021), suggesting its anticancer effect and potential prognostic roles. Jin et al. (Jin et al., 2021) stated that LINC00671 inhibited the progression of RCC by regulating the miR-221-5p/SOCS1 axis, and the overexpression of LINC00671 inhibited the proliferation, migration, invasion and EMT process of RCC cells *in vitro*. In addition, LINC00671 is capable of inhibiting pancreatic cancer cell proliferation and metastasis by suppressing the AKT and ERK signaling pathways (Qu et al., 2021a). These reports emphasized the inhibitory effect of LINC00671 on cell proliferation and metastasis, further supporting our results.

Finally, based on our risk stratification, we screened three drugs (lapatinib, axitinib, and CCT007093) as potential therapeutics for low-RILPS patients, while eight agents targeting the PI3K/MTOR signaling pathway or cell cycle were identified for high-RILPS patients. Notably, previous research has demonstrated that lapatinib, a dual tyrosine kinase inhibitor, can induce autophagic cell death and suppress HCC growth. Similarly, axitinib is a potent and selective inhibitor of vascular endothelial growth factor receptors (VEGFRs), and as an anti-angiogenic agent, it has shown promising efficacy against a variety of solid tumors, including advanced HCC. In addition, CCT007093 is a potent inhibitor of protein phosphatase 1D (PPM1D Wip1) that induces cell death by activating p38 kinase activity. For high-RILPS patients, the enrichment of PI3K/MTOR signaling and cell cycle pathways suggests higher proliferative and survival potential, making them more susceptible to agents targeting these pathways (Glaviano et al., 2023). The identification of these distinct therapeutic options underscores the importance of personalized medicine. Our findings provide valuable guidance for the selection of targeted therapies in both high- and low-RILPS patients, potentially improving clinical outcomes through precision treatment strategies.

To the best of our knowledge, this is the first risk score model for the prognostic characteristics of RRM-related LncRNAs established so far. The RILPS was found to be closely related to the pathological stage, immune microenvironment and prognosis of HCC, and may serve as an independent risk factor for HCC. The model demonstrated potential for prognostic stratification and individualized risk assessment. However, this study has several limitations that should be acknowledged. First, external validation using independent cohorts is lacking. In addition, although M-stage was identified as an independent prognostic factor and incorporated into the nomogram, only five patients in the TCGA-LIHC cohort were classified as M1. Therefore, conclusions regarding the prognostic contribution of M-stage and the corresponding nomogram component should be interpreted with caution, and further validation in larger cohorts with more metastatic cases is warranted. Second, the TCGA dataset used in this study is retrospective in nature, which may introduce inherent selection bias. Furthermore, the biological effects of LINC01138 were validated only in Huh7 cells. Validation in additional HCC cell lines would further strengthen the generalizability and robustness of these findings. Third, the sample size used for experimental validation was relatively limited, which may affect the robustness of the findings. In addition, although the RILPS model demonstrated favorable prognostic performance, its clinical translation may face challenges. Specifically, the complexity of the 65-lncRNA signature may limit its cost-effective application in routine clinical practice, and the stability of these lncRNAs across different HCC etiologies and patient populations requires further validation. Therefore, future studies with larger, prospective cohorts and simplified model optimization will be important to facilitate clinical application.

## Data Availability

The datasets presented in this study can be found in online repositories. The names of the repository/repositories and accession number(s) can be found in the article/Supplementary Material.
