# Supplementary material for: A prognostic lncRNA signature associated with ribonucleotide reductase predicts overall survival and immune landscape in hepatocellular carcinoma
Source: Front Cell Dev Biol. 2026 Aug 13;14:1908145. doi: 10.3389/fcell.2026.1908145 (PMC13518550; doi:10.3389/fcell.2026.1908145)
Supplement: Supplementary file 1 [file Table2.docx]

Table S2. Univariate Cox regression results of differential lncRNAs

|  | id | HR | L95CI | H95CI | pvalue |
| --- | --- | --- | --- | --- | --- |
| 1 | DYNLRB2.AS1 | 1.000842301 | 1.000052517 | 1.001632708 | 0.036586568 |
| 2 | C8orf44 | 1.002404851 | 1.000179208 | 1.004635448 | 0.034177695 |
| 3 | LINC02804 | 1.013283775 | 1.005770827 | 1.020852844 | 0.000510065 |
| 4 | SNHG27 | 1.002335673 | 1.001215342 | 1.003457257 | 4.34E-05 |
| 5 | KCNMB2.AS1 | 1.001550073 | 1.000370227 | 1.002731311 | 0.010010622 |
| 6 | LINC02177 | 1.001918568 | 1.00082786 | 1.003010465 | 0.000562614 |
| 7 | PSLNR | 1.009755577 | 1.001565918 | 1.018012201 | 0.019462755 |
| 8 | ZNF433.AS1 | 1.003237092 | 1.000239271 | 1.006243898 | 0.034289453 |
| 9 | LINC01730 | 1.017573701 | 1.002055799 | 1.033331915 | 0.02629057 |
| 10 | MIR583HG | 1.017792221 | 1.001581606 | 1.034265205 | 0.031327669 |
| 11 | LINC01579 | 1.006633149 | 1.001022156 | 1.012275594 | 0.020438794 |
| 12 | LINC02937 | 1.005292185 | 1.000884921 | 1.009718856 | 0.018545677 |
| 13 | ZFPM2.AS1 | 1.000561964 | 1.000173231 | 1.000950849 | 0.004602341 |
| 14 | LINC00399 | 1.011216357 | 1.00022682 | 1.022326637 | 0.045430846 |
| 15 | FAM83C.AS1 | 1.040214079 | 1.008554686 | 1.072867289 | 0.01241479 |
| 16 | LINC02932 | 1.004613061 | 1.002789849 | 1.006439588 | 6.84E-07 |
| 17 | SYBU.AS1 | 0.99796296 | 0.99596577 | 0.999964154 | 0.046039022 |
| 18 | LINC02803 | 1.02056372 | 1.002627344 | 1.038820967 | 0.024448777 |
| 19 | LINC01979 | 1.003863318 | 1.000562828 | 1.007174695 | 0.021742343 |
| 20 | WDFY3.AS2 | 1.002376373 | 1.000416522 | 1.004340064 | 0.017453868 |
| 21 | SLC12A5.AS1 | 1.013965025 | 1.003704658 | 1.02433028 | 0.007527382 |
| 22 | DLG5.AS1 | 1.003551933 | 1.000103791 | 1.007011963 | 0.043480468 |
| 23 | MVP.DT | 1.005474056 | 1.001002525 | 1.009965561 | 0.016368544 |
| 24 | CERS6.AS1 | 1.063134307 | 1.017003396 | 1.1113577 | 0.006832591 |
| 25 | MIR4733HG | 1.016954405 | 1.003298892 | 1.030795778 | 0.0147912 |
| 26 | LINC00967 | 1.006742437 | 1.000129899 | 1.013398695 | 0.045652088 |
| 27 | MIR4458HG | 1.000554526 | 1.000100907 | 1.001008351 | 0.016571532 |
| 28 | LINC01138 | 1.00027949 | 1.000025148 | 1.000533897 | 0.031257577 |
| 29 | LINC01203 | 1.001150458 | 1.000370311 | 1.001931213 | 0.003842242 |
| 30 | PTGES2.AS1 | 1.01087651 | 1.001925349 | 1.019907641 | 0.01713352 |
| 31 | LINC00671 | 0.998332742 | 0.996870568 | 0.99979706 | 0.02565667 |
| 32 | FOXD2.AS1 | 1.000730055 | 1.000111235 | 1.001349259 | 0.020755604 |
| 33 | DLX2.DT | 1.029112357 | 1.004412765 | 1.054419338 | 0.020602263 |
| 34 | CCDC13.AS1 | 1.002637897 | 1.000984698 | 1.004293827 | 0.001754499 |
| 35 | LINC01099 | 1.028633707 | 1.00172313 | 1.056267219 | 0.036865398 |
| 36 | MSC.AS1 | 1.000383624 | 1.000003371 | 1.000764022 | 0.048002674 |
| 37 | PFN2.AS1 | 1.118818783 | 1.012715891 | 1.236038143 | 0.027208046 |
| 38 | HECW2.AS1 | 1.002257352 | 1.000590119 | 1.003927364 | 0.007943141 |
| 39 | PUS1.AS1 | 1.017735481 | 1.004254824 | 1.031397096 | 0.009764986 |
| 40 | LINC01807 | 1.005003196 | 1.000938934 | 1.00908396 | 0.015783214 |
| 41 | TM4SF1.AS1 | 1.006464749 | 1.001419673 | 1.011535241 | 0.01196164 |
| 42 | SPTBN1.AS2 | 1.005510564 | 1.000992978 | 1.010048538 | 0.016758887 |
| 43 | LINC01169 | 1.004939051 | 1.000227204 | 1.009673094 | 0.039907382 |
| 44 | LARP4B.DT | 1.050352957 | 1.006261937 | 1.096375897 | 0.024750976 |
| 45 | DUXAP8 | 1.002550141 | 1.001319413 | 1.003782382 | 4.83E-05 |
| 46 | LINC01929 | 1.001604311 | 1.000167305 | 1.003043382 | 0.028645038 |
| 47 | PKIA.AS1 | 1.002372287 | 1.000147299 | 1.004602225 | 0.03662929 |
| 48 | LINC01980 | 1.000875561 | 1.0002084 | 1.001543167 | 0.010097742 |
| 49 | KLHDC7B.DT | 1.036151754 | 1.015146211 | 1.057591947 | 0.000677461 |
| 50 | LINC02274 | 1.009526352 | 1.001437499 | 1.017680541 | 0.020891899 |
| 51 | LINC01193 | 1.004141046 | 1.001091089 | 1.007200295 | 0.007754633 |
| 52 | ARHGEF2.AS2 | 1.008962261 | 1.000253694 | 1.017746647 | 0.043662521 |
| 53 | LINC02466 | 1.008644708 | 1.000910874 | 1.0164383 | 0.028393911 |
| 54 | MIR663AHG | 1.001941784 | 1.000485552 | 1.003400135 | 0.008946023 |
| 55 | LINC01885 | 1.022082995 | 1.01086746 | 1.033422967 | 0.000104476 |
| 56 | DLGAP1.AS3 | 1.004262275 | 1.001523198 | 1.007008843 | 0.002271567 |
| 57 | CASC20 | 1.00552587 | 1.001565347 | 1.009502055 | 0.006204892 |
| 58 | LINC01428 | 0.980125251 | 0.962126308 | 0.998460909 | 0.033767086 |
| 59 | LINC02930 | 1.034503842 | 1.009884123 | 1.05972376 | 0.005774649 |
| 60 | NOVA1.DT | 1.006078929 | 1.002235995 | 1.009936599 | 0.001910383 |
| 61 | HOXD.AS2 | 1.011892375 | 1.002269609 | 1.02160753 | 0.015309235 |
| 62 | LINC00668 | 1.002199797 | 1.000433151 | 1.003969563 | 0.014644996 |
| 63 | LINC02293 | 1.008712479 | 1.001130153 | 1.016352232 | 0.024235888 |
| 64 | LINC00322 | 1.003536346 | 1.001984733 | 1.005090361 | 7.77E-06 |
| 65 | WARS2.IT1 | 1.02204037 | 1.0039976 | 1.040407386 | 0.016440662 |
| 66 | LINC00858 | 1.012404256 | 1.004020481 | 1.020858039 | 0.003664468 |
| 67 | LINC01150 | 1.021277915 | 1.002733304 | 1.040165492 | 0.02432823 |
| 68 | FSIP2.AS1 | 1.029079132 | 1.005399634 | 1.053316338 | 0.015806385 |
| 69 | LINC02641 | 1.021670705 | 1.008748616 | 1.034758326 | 0.000962653 |
| 70 | LINC00624 | 1.000495588 | 1.00007823 | 1.000913121 | 0.019942222 |
| 71 | WASIR2 | 1.032118366 | 1.014566557 | 1.049973819 | 0.000303266 |
| 72 | LINC01907 | 1.133772986 | 1.050864436 | 1.223222653 | 0.001193276 |
| 73 | KCNQ1.AS1 | 1.047584244 | 1.016048603 | 1.080098674 | 0.002874204 |
| 74 | LINC02211 | 1.004504303 | 1.001099532 | 1.007920653 | 0.009477377 |
| 75 | LINC00559 | 1.079288463 | 1.02544868 | 1.135955029 | 0.003472391 |
| 76 | LINC01901 | 1.016153743 | 1.003793827 | 1.02866585 | 0.010275727 |
| 77 | FXYD6.AS1 | 1.002505962 | 1.000288969 | 1.004727869 | 0.026708559 |
| 78 | LINC02228 | 1.00136757 | 1.000477201 | 1.002258732 | 0.002602666 |
| 79 | LINC02405 | 1.006621403 | 1.001993703 | 1.011270476 | 0.004998261 |
| 80 | CT62 | 1.034995051 | 1.000951996 | 1.070195932 | 0.043828559 |
| 81 | GOLGA7B.DT | 1.057859289 | 1.003428719 | 1.115242423 | 0.036891454 |
| 82 | CNIH3.AS1 | 1.01360725 | 1.004355052 | 1.02294468 | 0.003867321 |
| 83 | FAM225B | 1.014446459 | 1.003064812 | 1.025957253 | 0.012719149 |
| 84 | UCA1 | 1.000242265 | 1.000135464 | 1.000349078 | 8.74E-06 |
| 85 | POU2F2.AS2 | 1.000285754 | 1.000016632 | 1.000554949 | 0.037424141 |
| 86 | RCAN2.DT | 1.020516196 | 1.000525639 | 1.040906166 | 0.044217107 |
| 87 | LINC01416 | 1.022762899 | 1.011500768 | 1.034150424 | 6.77E-05 |
| 88 | HOXC.AS1 | 1.00876501 | 1.000224226 | 1.017378722 | 0.044257348 |
| 89 | LINC02462 | 1.094912968 | 1.042653246 | 1.149792045 | 0.000279187 |
| 90 | FAM182A | 1.015606491 | 1.007168507 | 1.024115167 | 0.000274764 |
| 91 | LINC02391 | 1.106526509 | 1.056955411 | 1.158422485 | 1.50E-05 |
| 92 | FAM88E | 1.079934308 | 1.009017791 | 1.155835032 | 0.026485857 |
| 93 | LINC02327 | 1.003597218 | 1.000563505 | 1.006640129 | 0.020089252 |
| 94 | LINC00689 | 1.00137424 | 1.000030212 | 1.002720074 | 0.045064559 |
| 95 | LINC02820 | 1.009388613 | 1.000655705 | 1.018197735 | 0.035046881 |
| 96 | LINC01139 | 1.000238309 | 1.000036513 | 1.000440146 | 0.020632583 |
| 97 | FBXO34.AS1 | 1.138210292 | 1.038601249 | 1.247372531 | 0.005596535 |
| 98 | LINC00676 | 1.026772393 | 1.007637369 | 1.046270791 | 0.00591137 |
| 99 | FAM85B | 1.033636902 | 1.005772592 | 1.062273175 | 0.017654274 |
| 100 | LINC03068 | 1.002893388 | 1.001343571 | 1.004445602 | 0.000250686 |
| 101 | LINC00879 | 1.001796156 | 1.000698111 | 1.002895406 | 0.00134042 |
| 102 | G2E3.AS1 | 1.004386223 | 1.000095824 | 1.008695028 | 0.045087772 |
| 103 | LINC01163 | 1.007814447 | 1.000620526 | 1.015060089 | 0.033197683 |
| 104 | LINC01019 | 1.002319311 | 1.000671823 | 1.003969511 | 0.00577771 |
| 105 | LINC00221 | 1.000824341 | 1.000287102 | 1.001361868 | 0.002631397 |
| 106 | RNF157.AS1 | 1.001734352 | 1.000386433 | 1.003084088 | 0.011657352 |
| 107 | CLYBL.AS2 | 1.095394439 | 1.004463751 | 1.194556773 | 0.039332254 |
| 108 | ROCR | 1.004969711 | 1.000138452 | 1.009824309 | 0.043771625 |
| 109 | FAM3D.AS1 | 1.01652242 | 1.000539564 | 1.032760589 | 0.042695622 |
| 110 | DNAH17.AS1 | 1.013203034 | 1.000115438 | 1.026461896 | 0.048000321 |
| 111 | LINC01841 | 1.024292292 | 1.008734937 | 1.040089584 | 0.002114135 |
| 112 | DSCR4 | 1.00671798 | 1.002148765 | 1.011308028 | 0.003917023 |
| 113 | MIR4300HG | 1.021404506 | 1.001798053 | 1.041394681 | 0.03222382 |
| 114 | LINC01194 | 1.003716279 | 1.001677268 | 1.00575944 | 0.000349958 |
| 115 | GPC6.AS1 | 1.006824642 | 1.001644681 | 1.012031391 | 0.009755156 |
| 116 | PBX3.DT | 1.10881083 | 1.033610267 | 1.189482628 | 0.003944963 |
| 117 | FSIP2.AS2 | 1.009734723 | 1.002787319 | 1.016730259 | 0.005957223 |
| 118 | MIR4422HG | 1.134055364 | 1.037112774 | 1.240059521 | 0.005793672 |
| 119 | DSCR8 | 1.001794392 | 1.000535386 | 1.003054982 | 0.005203195 |
| 120 | LINC01705 | 1.003169612 | 1.000307641 | 1.006039771 | 0.029932728 |
| 121 | LINC01242 | 1.016171889 | 1.004973942 | 1.027494608 | 0.004545946 |
| 122 | TMEM18.DT | 1.004599588 | 1.001710829 | 1.007496677 | 0.001787788 |
| 123 | PPM1B.DT | 1.094968954 | 1.004846111 | 1.193174752 | 0.038426084 |
| 124 | FLJ16779 | 1.000981051 | 1.000168277 | 1.001794484 | 0.017983572 |
| 125 | LINC01287 | 1.000090464 | 1.000017802 | 1.000163131 | 0.014679822 |
| 126 | LINC01361 | 1.00064097 | 1.000138482 | 1.00114371 | 0.012409129 |
| 127 | LINC02835 | 1.003816745 | 1.00056997 | 1.007074056 | 0.021183889 |
| 128 | NBAT1 | 1.053126317 | 1.010418419 | 1.097639373 | 0.014259526 |
| 129 | MAFA.AS1 | 1.000637532 | 1.000052153 | 1.001223254 | 0.032790708 |
| 130 | LINC02267 | 1.009158618 | 1.002114849 | 1.016251896 | 0.010737699 |
| 131 | LINC02373 | 1.117690125 | 1.045219859 | 1.195185114 | 0.001141774 |
| 132 | LINC01192 | 1.016296796 | 1.004251983 | 1.028486073 | 0.007872803 |
| 133 | LINC01967 | 1.015765589 | 1.004156483 | 1.027508908 | 0.007648252 |
| 134 | LINC01910 | 1.017902687 | 1.00675356 | 1.029175284 | 0.00158969 |
| 135 | HAS2.AS1 | 1.010685573 | 1.00022016 | 1.021260487 | 0.04534764 |
| 136 | LINC01756 | 1.119094824 | 1.012732328 | 1.236628072 | 0.02722581 |
| 137 | LMO7DN | 1.062823761 | 1.009557199 | 1.118900791 | 0.020203797 |
| 138 | ST8SIA6.AS1 | 1.000596303 | 1.000190831 | 1.001001939 | 0.003943136 |
| 139 | LINC03044 | 1.007523231 | 1.000016915 | 1.01508589 | 0.049484042 |
| 140 | LINC01721 | 1.00415799 | 1.000959623 | 1.007366577 | 0.010795675 |
| 141 | ROR1.AS1 | 1.002178785 | 1.000018415 | 1.004343823 | 0.048076838 |
| 142 | TFPI2.DT | 1.044702974 | 1.014763346 | 1.075525941 | 0.003200217 |
| 143 | LINC02315 | 1.002662319 | 1.000697465 | 1.00463103 | 0.007892847 |
| 144 | LINC02806 | 1.009147238 | 1.002587777 | 1.015749614 | 0.006205508 |
| 145 | LINC01611 | 1.006937696 | 1.0014524 | 1.012453036 | 0.013111649 |
| 146 | TMEM132E.DT | 1.149855713 | 1.025166556 | 1.289710585 | 0.017107912 |
| 147 | LINC02444 | 1.017631016 | 1.003597975 | 1.031860277 | 0.013628851 |
| 148 | LINC01764 | 1.087854738 | 1.03953462 | 1.138420894 | 0.000280614 |
| 149 | GATA3.AS1 | 1.065425549 | 1.015339239 | 1.117982597 | 0.009891664 |
| 150 | LINC01793 | 1.006803292 | 1.000958607 | 1.012682105 | 0.022458867 |
| 151 | LINC01363 | 1.207455007 | 1.068957086 | 1.363897217 | 0.002423478 |
| 152 | DSCR4.IT1 | 1.011360574 | 1.000339818 | 1.022502746 | 0.043306593 |
| 153 | MIR548XHG | 1.001770773 | 1.000305467 | 1.003238225 | 0.017840482 |
